# Early-Life Gut Microbiota and the Pediatric Gut–Lung Axis: A Systematic Review of Clinical, Preclinical, and Mechanistic Evidence

**DOI:** 10.3390/nu18193260

**Published:** 2026-10-02

**Authors:** Salvatore Michele Carnazzo, Alessandro Vitello, Andrea Domenico Praticò, Marcello Maida, Vassilios Fanos

**Affiliations:** 1 Faculty of Medicine and Surgery, Kore University of Enna, 94100 Enna, Italy; alessandro.vitello@unikore.it (A.V.); andrea.pratico@unikore.it (A.D.P.); marcello.maida@unikore.it (M.M.); 2 Neonatal Intensive Care Unit, Department of Surgical Sciences, University of Cagliari, 09124 Cagliari, Italy; vafanos@jpnim.com

**Keywords:** gut–lung axis, gut microbiota, pediatric respiratory health, early life, short-chain fatty acids, probiotics, immune development

## Abstract

**Background/Objectives:** The early-life gut microbiota plays a pivotal role in immune maturation and may influence respiratory health through the gut–lung axis. However, evidence linking microbial development, dysbiosis, microbial metabolites, and microbiota-targeted interventions to pediatric respiratory outcomes remains heterogeneous. This systematic review aimed to synthesize clinical and preclinical evidence on the pediatric gut–lung axis and its underlying mechanisms. **Methods:** This systematic review followed PRISMA 2020 guidelines and a prospectively registered PROSPERO protocol (CRD420251062905). PubMed, Scopus, and Web of Science were searched for studies published from January 2014 to March 2025. Eligible primary studies included randomized controlled trials, observational studies, and mechanistically relevant animal models. Risk of bias was assessed using RoB 2, the Newcastle–Ottawa Scale, and SYRCLE’s tool, respectively. Reviews were examined separately to provide mechanistic and translational context without contributing to the primary evidence synthesis. **Results:** Twenty-five primary studies (11 clinical and 14 preclinical) were included in the systematic evidence synthesis, while 54 reviews provided complementary contextual evidence. Early gut and airway microbial communities showed substantial overlap after birth followed by progressive niche-specific divergence. Early-life dysbiosis, including depletion of beneficial taxa and expansion of potentially pathogenic bacteria, was associated with asthma, recurrent wheezing, respiratory infections, and neonatal lung injury. Short-chain fatty acids emerged as key mediators of gut–lung immune signaling. Clinical trials suggested potential respiratory and immunological benefits of probiotic and synbiotic interventions, while preclinical models supported mechanistic links involving intestinal barrier dysfunction, microbial metabolites, and inflammatory pathways. **Conclusions:** Current evidence supports a biologically plausible and clinically relevant pediatric gut–lung axis. However, methodological heterogeneity and the limited availability of longitudinal clinical studies warrant cautious interpretation and further well-designed research before microbiota-targeted strategies can be routinely translated into pediatric respiratory care.

## 1. Introduction

Microbial ecosystems play a central role in regulating immune maturation, metabolic programming, and mucosal homeostasis [1]. Rather than functioning as isolated ecological niches, microbial communities distributed across different anatomical sites participate in interconnected networks of microbial, metabolic, and immunological signaling. Among these, the gut–lung axis has emerged as a bidirectional interface through which intestinal microbial communities and their metabolites may influence respiratory immunity and pulmonary homeostasis, while respiratory disease and inflammation may, in turn, affect intestinal microbial ecology.

This interaction is particularly relevant during early life, when the gut microbiota, immune system, and epithelial barriers undergo rapid and coordinated development [1]. From birth through childhood, microbial colonization is shaped by multiple environmental and nutritional determinants, including mode of delivery, breastfeeding and formula feeding, antibiotic exposure, diet, and environmental microbial diversity [2]. These exposures may influence the establishment of beneficial microbial communities and the production of bioactive microbial metabolites, thereby contributing to immune tolerance or, conversely, inflammatory dysregulation.

Mechanistic evidence supports a biological connection between intestinal microbial activity and pulmonary immunity. Gut-derived metabolites, particularly short-chain fatty acids (SCFAs), can modulate distal mucosal immune responses, promote regulatory T-cell differentiation, and suppress inflammatory pathways within the respiratory tract [3]. Conversely, intestinal dysbiosis and impaired gut barrier integrity have been associated with systemic inflammation, altered immune signaling, and increased susceptibility to pulmonary injury [4]. These observations provide a mechanistic framework through which early microbial and nutritional exposures may influence subsequent respiratory health.

Clinical evidence has increasingly supported the relevance of these mechanisms in pediatric populations. Altered early-life microbial profiles have been associated with bronchiolitis, recurrent wheezing, asthma, respiratory infections, and bronchopulmonary dysplasia (BPD) [5]. In preterm infants, aberrant microbial colonization has been linked to systemic immune activation and adverse neonatal outcomes [6]. In older children, specific microbial signatures have also been associated with asthma phenotypes, allergic sensitization, and persistent alterations in immune development [7]. Furthermore, microbiota-targeted nutritional strategies, including probiotics, prebiotics, synbiotics, and dietary modulation, have attracted increasing interest as potential approaches to influence gut microbial composition and downstream respiratory immunity.

Despite this growing body of evidence, important uncertainties remain. Available studies differ substantially in population characteristics, biological samples, sequencing methodologies, microbial endpoints, respiratory outcomes, and intervention strategies. Moreover, associations observed in human cohorts coexist with mechanistic evidence derived largely from experimental models, complicating the assessment of causality and clinical translation. Existing literature therefore remains fragmented across microbiology, nutrition, immunology, neonatology, and pediatric respiratory medicine, limiting the development of an integrated developmental framework for the pediatric gut–lung axis.

Accordingly, this systematic review aimed to synthesize and critically appraise clinical and preclinical evidence linking early-life gut microbiota development with pediatric respiratory health, with particular attention to microbial colonization, dysbiosis, microbiota-derived metabolites, environmental and nutritional determinants, and microbiota-targeted interventions. The available evidence supports a biologically plausible gut–lung axis in early life, while also highlighting substantial methodological heterogeneity and the need for well-designed longitudinal and interventional studies before microbiota-targeted strategies can be routinely incorporated into pediatric respiratory care.

## 2. Materials and Methods

### 2.1. Study Design

This systematic review was conducted in accordance with the Preferred Reporting Items for Systematic Reviews and Meta-Analyses (PRISMA) 2020 guidelines [8,9]. The aim was to synthesize and critically appraise current evidence on the role of early-life gut microbiota and its contribution to respiratory health across infancy and childhood, through the gut-lung axis. In parallel, secondary literature, including systematic, scoping, and narrative reviews, was screened separately to provide complementary mechanistic and translational context. These publications were not included in the primary evidence synthesis or outcome analysis. Instead, these reviews were used to provide additional mechanistic and conceptual context particularly in areas where primary data were sparse or fragmented. This approach broadened the conceptual coverage of the pediatric gut–lung axis while preserving the methodological distinction between the systematic synthesis of primary studies and the complementary narrative interpretation of secondary evidence. The protocol for this systematic review was prospectively registered in PROSPERO (International Prospective Register of Systematic Reviews) under ID CRD420251062905. The full protocol is available at: https://www.crd.york.ac.uk/PROSPERO/view/CRD420251062905 (accessed on 27 September 2026).

### 2.2. Eligibility Criteria

Eligibility criteria were defined using the PICOS framework and comprised the following elements:

- Population: Infants and children aged 0 to 16 years, representing the critical period before microbiota stabilization and immune system maturation.
- Interventions/Exposures: Early-life microbial colonization patterns; perinatal factors influencing gut microbiota (e.g., delivery mode, feeding, antibiotic exposure, environmental exposures); microbiota-targeted interventions (e.g., probiotics, prebiotics, dietary modulation).
- Comparators: Healthy controls, alternative microbial profiles, or environmental exposures, when applicable.
- Outcomes: Respiratory outcomes (e.g., asthma, bronchiolitis, wheezing, respiratory infections, bronchopulmonary dysplasia), and immunological correlates (e.g., cytokine profiles, T-regulatory cell induction, mucosal immune parameters).

### 2.3. Study Types

Primary studies eligible for the systematic evidence synthesis included:

- Clinical studies (randomized controlled trials [RCTs], cohort studies, case–control studies, and longitudinal studies);
- Preclinical studies using animal models with translational relevance to pediatric development.

Systematic, scoping, and narrative reviews were screened separately and retained only to provide complementary mechanistic and translational context. These secondary publications were not included in the primary evidence synthesis or outcome analysis.

The complete characteristics and bibliographic details of the 54 secondary reviews retained for contextual analysis are provided in Supplementary Table S1.

### 2.4. Exclusion Criteria

- Studies focused on adults or adolescents > 16 years.
- Studies reporting only maternal microbiota without infant/neonatal data.
- Case reports, editorials, commentaries, and opinion pieces without primary data.
- In vitro studies without in vivo validation.
- Animal models without translational relevance to pediatric human biology.
- Studies unrelated to the gut–lung axis (e.g., gut–brain).
- Non-peer-reviewed materials (preprints, conference abstracts without full data).
- Non-English publications without available English abstract or translation.
- Articles published before 2014, unless considered seminal for background reference.

### 2.5. Supplementary Targeted Literature Search

The predefined systematic search required the simultaneous presence of multiple concept blocks and therefore preferentially retrieved studies explicitly combining pediatric populations, respiratory outcomes, perinatal determinants, immune mechanisms, and microbiota-targeted interventions. To address the resulting risk of incomplete conceptual coverage, a supplementary targeted literature search was conducted in PubMed and updated on 9 September 2026. The principal supplementary PubMed search used the following query: ((“gut microbiota”[Title/Abstract] OR “gut microbiome”[Title/Abstract]) AND (“gut-lung axis”[Title/Abstract] OR “gut lung axis”[Title/Abstract]) AND (infant*[Title/Abstract] OR child*[Title/Abstract] OR pediatric*[Title/Abstract] OR neonat*[Title/Abstract])). This search retrieved 96 records, which were screened for relevance to the contextual and mechanistic scope of the review. Additional domain-specific searches and citation tracking addressed environmental microbial exposures, short-chain fatty acids, regulatory T cells, airway epithelial function, epigenetic regulation, and human-milk bioactive compounds. Records were considered for contextual inclusion when they provided direct pediatric evidence, developmentally relevant preclinical evidence, or essential mechanistic information related to the gut–lung axis. General microbiome publications, adult-only clinical studies, studies unrelated to respiratory outcomes, and redundant reviews were not retained. Evidence identified through this supplementary approach was used to expand the Introduction and Discussion and was not retrospectively incorporated into the predefined systematic study counts, Table 1 and Table 2, PRISMA flow diagram, or risk-of-bias assessment.

### 2.6. Information Sources and Search Strategy

The predefined systematic search strategy was developed in accordance with PRISMA 2020 recommendations. We searched PubMed, Web of Science, and Scopus for peer-reviewed articles published between 1 January 2014, and 31 March 2025 (last search performed on 31 March 2025). A single predefined search string was developed in PubMed and applied to Web of Science and Scopus with minor syntax adjustments (e.g., field tags and truncation symbols), without conceptual modifications. The search strategy combined controlled vocabulary (e.g., MeSH terms in PubMed) and free-text keywords related to gut microbiota, respiratory health, immune development, and pediatric populations. Boolean operators and truncation were used to structure the search. The core search terms were grouped as follows: (“Gut Microbiota” OR “Intestinal Microbiome” OR “Infant Microbiota”) AND (“Lung” OR “Pulmonary” OR “Respiratory Tract” OR “Airway Microbiome”) AND (“Infant” OR “Child” OR “Pediatric” OR “Neonatal”) AND (“Immune Development” OR “Cytokines” OR “Inflammation” OR “Toll-like Receptors”) AND (“Breastfeeding” OR “Cesarean Section” OR “Antibiotics”) AND (“Asthma” OR “Bronchiolitis” OR “Wheezing” OR “Respiratory Infection”) AND (“Probiotics” OR “Prebiotics” OR “Microbiota Modulation”).

The full search string and database-specific syntax are provided in Supplementary Material File S1 to ensure reproducibility.

### 2.7. Study Selection Process

**Figure 1:**
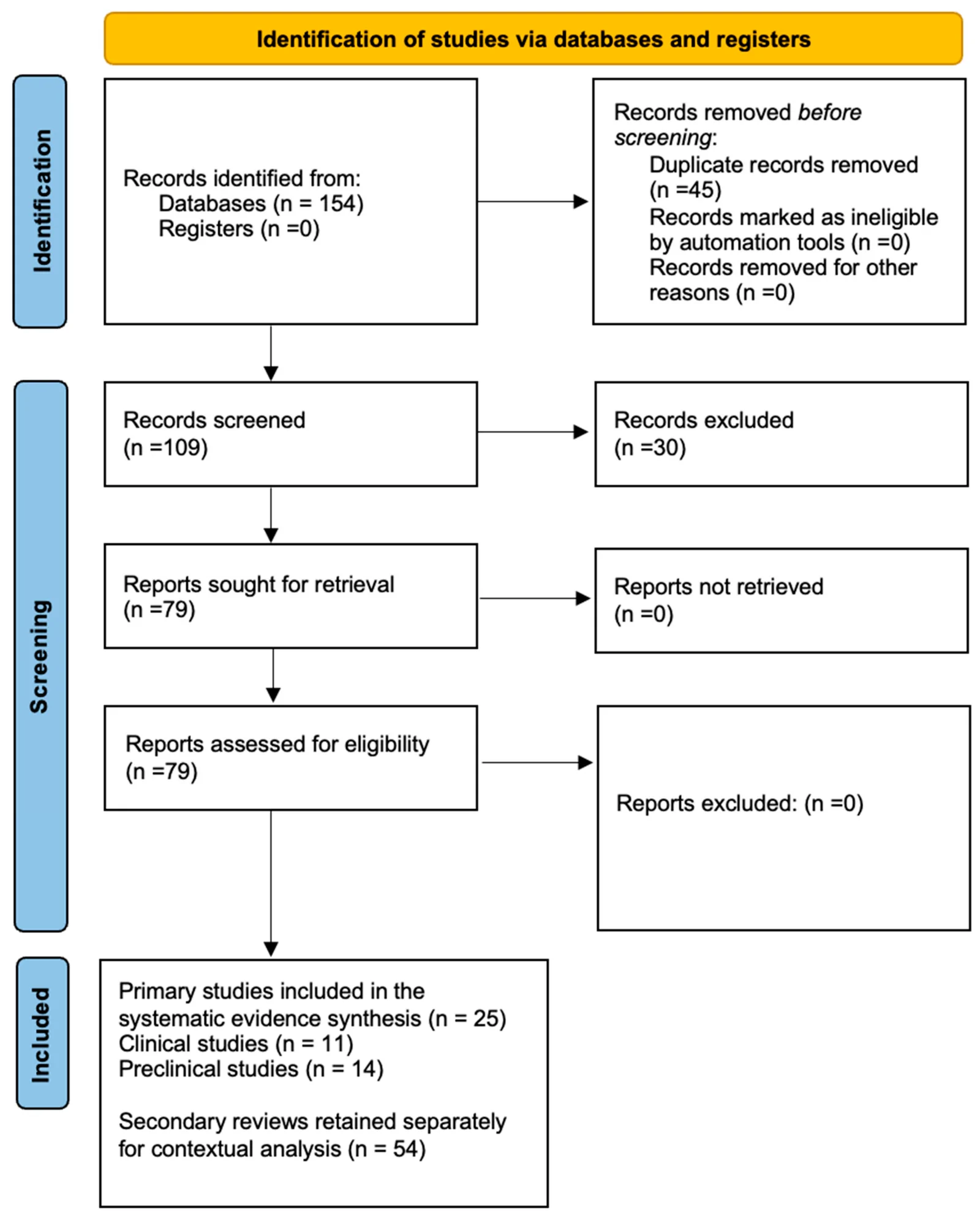
PRISMA 2020 flow diagram of the study-selection process. Of the 79 publications retained after full-text assessment, 25 primary studies were included in the systematic evidence synthesis and 54 secondary reviews were retained separately for complementary contextual analysis. Secondary reviews were not incorporated into the primary evidence synthesis or risk-of-bias assessment [8].

All retrieved records were exported to Rayyan QCRI for systematic screening. Duplicate records were identified and removed using Rayyan’s automated deduplication tool and manual verification. Two independent reviewers (S.C. and D.B.) screened titles and abstracts against the predefined eligibility criteria. Full texts were then evaluated for final inclusion. Disagreements were resolved by consensus or adjudicated by a third reviewer (A.V.). Secondary reviews meeting the predefined contextual eligibility criteria were cataloged separately and were not included in the primary evidence synthesis. The study selection process was documented using a PRISMA 2020 flow diagram (Figure 1), summarizing the number of records identified, screened, excluded, and included at each stage of the systematic review.

A standardized data extraction template was developed and piloted on a subset of studies to ensure consistency. Extracted data included:

- Author, year, country of origin.
- Study design and type (clinical, preclinical, review).
- Population characteristics (age, number of participants, clinical condition).
- Microbiota analysis method (e.g., 16S rRNA, shotgun metagenomics, culture).
- Exposure variables (delivery mode, antibiotic use, diet, etc.).
- Immune and respiratory outcomes.
- Microbiota-related findings (taxonomic shifts, SCFA levels, dysbiosis indices).
- Reported mechanistic insights (e.g., immune pathways, epithelial signaling).

### 2.8. Risk of Bias and Quality Assessment

Risk of bias was assessed independently by two reviewers using tools appropriate to study design:

- RCT: Cochrane Risk of Bias 2.0 (RoB 2) [35].
- Observational studies: Newcastle–Ottawa Scale (NOS) [36].
- Animal studies: SYRCLE’s Risk of Bias tool [37].

**Secondary reviews** were **retained** exclusively **for contextual** and mechanistic interpretation and **were not incorporated into the** formal systematic synthesis or **risk-of-bias** assessment. **AMSTAR 2** is designed for the critical appraisal of systematic reviews that include randomized or non-randomized studies of healthcare interventions [38]. Given the heterogeneous types and exclusively contextual role of the secondary **reviews** retained in this study, no formal review-level **methodological** appraisal was undertaken. For observational studies, NOS domain scores (0–9) were calculated and categorized as low (7–9), moderate (4–6), or high (0–3) risk of bias [36].

For randomized controlled trials, the Cochrane Risk of Bias 2 tool was applied, and domain-level assessments were used to generate the prescribed categorical judgments of low risk of bias, some concerns, or high risk of bias, without assigning numerical scores [35].

For animal studies, the SYRCLE’s Risk of Bias tool was used to evaluate core methodological domains (e.g., sequence generation, allocation concealment, random housing, blinding, outcome reporting), and overall risk was classified into low, moderate, or high [37]. When randomization, allocation, housing, or blinding procedures were not explicitly reported, the corresponding SYRCLE domains were classified as unclear risk of bias rather than being left unassessed. Overall judgments were assigned qualitatively on the basis of the distribution and potential influence of domain-level concerns, as the SYRCLE tool does not prescribe a numerical score or a standardized algorithm for deriving an overall rating.

Discrepancies were resolved by consensus. Risk of bias assessments informed the interpretation of study quality and weighting of evidence in the narrative synthesis.

Due to heterogeneity in study designs, outcomes, and methodologies, meta-analysis was not performed. Instead, studies were synthesized within thematic domains:

1. Microbiota composition and immune programming;
2. Dysbiosis and respiratory disease onset;
3. Environmental and perinatal modulators (e.g., antibiotics, delivery mode);
4. Therapeutic modulation (e.g., probiotics, prebiotics, nutritional strategies).

The main characteristics of the primary studies included in the systematic evidence synthesis are presented separately according to study type: clinical studies are summarized in Table 1, whereas preclinical studies are summarized in Table 2.

Detailed risk-of-bias assessments for each included study are provided in Supplementary Table S2 (Newcastle–Ottawa Scale for observational studies), Supplementary Table S3 (SYRCLE’s Risk of Bias tool for animal studies), and Supplementary Table S4 (Cochrane RoB 2 for randomized controlled trials). Raw scoring sheets are provided as Excel files to ensure transparency and reproducibility.

The interpretation of the findings considered the design-specific risk-of-bias assessments. Studies presenting substantial methodological concerns were given limited interpretative weight and did not drive the principal conclusions.

## 3. Results

### 3.1. Study Selection

The study-selection process is summarized in Figure 1. Overall, 25 primary studies, comprising 11 clinical and 14 preclinical studies, were included in the systematic evidence synthesis, while 54 secondary reviews were retained separately for complementary mechanistic and contextual interpretation. The secondary reviews are listed in Supplementary Table S1, whereas the characteristics of the primary clinical and preclinical studies are presented in Table 1 and Table 2, respectively.

This table summarizes human studies, including randomized controlled trials, cohort studies, and observational designs, examining the relationship between early-life gut microbiota and respiratory outcomes. Reported outcomes include respiratory tract infections, asthma incidence, lung function parameters, and immune modulation. Microbiota-targeted interventions such as probiotics and synbiotics are also highlighted.

Abbreviations: CF, cystic fibrosis; FEV1, forced expiratory volume in 1 s; LRTI, lower respiratory tract infection; RCT, randomized controlled trial; RTI, respiratory tract infection; SCFA, short-chain fatty acid.

This table summarizes experimental studies conducted in animal models, including mice, rats, and calves, exploring the mechanistic links between gut microbiota alterations and respiratory outcomes. Key investigated pathways include microbial dysbiosis, short-chain fatty acid signaling, immune modulation, and inflammatory responses in lung tissue. These studies provide mechanistic insights supporting the biological plausibility of the gut–lung axis in respiratory disease development.

Abbreviations: AMP, antimicrobial peptide; BPD, bronchopulmonary dysplasia; CF, cystic fibrosis; SCFA, short-chain fatty acid; SFB, segmented filamentous bacteria; TCM, traditional Chinese medicine.

### 3.2. Early-Life Microbiota Colonization and Cross-Niche Dynamics

Early-life microbial colonization is initially characterized by cross-niche overlap between gut, oral, and airway communities, which diverges rapidly during the first months of life, shaping immune compartmentalization and respiratory susceptibility.

In a large longitudinal cohort of 112 term infants, Reyman et al. (2021) [18] profiled 1248 samples across feces, saliva, and nasopharynx, reporting a decline in shared operational taxonomic units (OTUs) from 57.5% at week 1 to 29.0% at 6 months (*p* < 0.001). This shift was characterized by reduced abundance of *Dolosigranulum* and *Corynebacterium* and increased representation of potential pathogens such as *Haemophilus* and *Neisseria meningitidis*, indicating ecological divergence between mucosal sites.

Wang et al. (2023) [17] confirmed this pattern in a smaller cohort of 20 neonates, showing early overlap between gut and pharyngeal microbiota dominated by *Bifidobacterium* and *Escherichia-Shigella*, which rapidly shifted to *Streptococcus* (38.4%) and *Staphylococcus* (23.1%) in the oropharynx. Predictive functional profiling revealed niche-specific enrichment of immune-modulatory pathways. In preterm infants, Yang et al. (2021) [13] observed overlapping colonization by *Ureaplasma* and *Fusobacterium* at birth, followed by accelerated divergence by day 28, suggesting that prematurity modifies early microbial trajectories and mucosal immune imprinting. Collectively, these findings highlight early cross-niche microbial divergence as a potential determinant of airway colonization and immune programming, setting the stage for downstream respiratory vulnerability.

### 3.3. Gut Dysbiosis and Systemic Inflammatory Conditioning of the Lung

Human observational studies and mechanistic models suggest that intestinal dysfunction and early-life microbial perturbations may be associated with systemic inflammatory conditioning and increased respiratory susceptibility. In a cohort of 20,287 children, Huang et al. (2021) [14] reported that chronic constipation was associated with an increased subsequent risk of asthma (adjusted hazard ratio [aHR], 2.36; 95% confidence interval [CI], 2.04–2.73; *p* < 0.001), with a stronger association observed among laxative users. However, microbiota composition, microbial metabolites, and intestinal inflammatory biomarkers were not directly assessed in that study.

This epidemiological association was subsequently contextualized using evidence from animal experiments. Alhasan et al. (2023) [28] demonstrated that maternal antibiotic exposure impaired SCFA synthesis, increased intestinal permeability, assessed using the fluorescein isothiocyanate–dextran (FITC-dextran) assay (*p* < 0.001), and elevated serum LPS levels. Offspring exhibited an altered T-helper 17/regulatory T-cell (Th17/Treg) balance (increased RORγt-positive and decreased Foxp3-positive cells), heightened pulmonary IL-6 and IL-17, and alveolar simplification. In complementary work, Wedgwood et al. (2020) [34] found that growth restriction and hyperoxia promoted *Enterobacteriaceae* overgrowth, enhanced TLR4/NF-κB activation, and induced pulmonary hypertension and BPD phenotypes, effects reversed by TLR4 blockade. Willis et al. (2020) [24] further showed that perinatal antibiotic exposure reduced *Lactobacillus*, increased *Peptostreptococcaceae*, suppressed IL-22, and impaired epithelial regeneration, resulting in pulmonary fibrosis and vascular rarefaction.

Collectively, these findings indicate that early-life gut dysbiosis functions as a systemic inflammatory amplifier, driving pulmonary immune activation and increasing vulnerability to chronic respiratory disease.

### 3.4. Microbial Metabolites and Short-Chain Fatty Acids in Immune Regulation

SCFAs act as key immunometabolites in gut-lung crosstalk. Depner et al. [15] found that farm-exposed children had elevated fecal butyrate and a 72% reduction in asthma risk (odds ratio [OR] = 0.28, *p* = 0.034). Microbial maturity scores were higher (β = 0.27, *p* = 0.001), suggesting accelerated immunological education. Papotto et al. (2023) [26] revealed that γδ T cell-deficient dams yielded offspring with reduced pentanoate and hexanoate, higher pulmonary IL-4, IL-5, IL-33, and enhanced group 2 innate lymphoid cell (ILC2) activity. SCFA supplementation normalized cytokine levels and airway eosinophilia. Price et al. (2024) [21] supplemented CF mice with *Bacteroides*, raising propionate, lowering *Escherichia*/*Shigella*, and reducing interleukin-6 (IL-6), macrophage inflammatory protein-1β (MIP-1β), and granulocyte colony-stimulating factor (G-CSF) concentrations in bronchoalveolar lavage fluid (BALF). Liu et al. (2020) [29] linked alanylglutamine-driven butyrate elevation to GPR43 activation and reduced IL-1β, IL-6, and transforming growth factor beta (TGF-β) in bronchoalveolar tissue. Together, these findings underscore the central role of SCFAs as modulators of airway inflammation and immune programming, linking gut microbial activity to respiratory outcomes through metabolite-mediated signaling.

### 3.5. Probiotics and Synbiotics: Modulators of Respiratory Immunity

Multiple randomized controlled trials have demonstrated that probiotic and synbiotic interventions can modulate respiratory immunity through effects on microbial composition, immune mediators, and clinical outcomes. Sjödin et al. (2023) [10] randomized infants to synbiotic (*L. paracasei* F19 + prebiotics) or control, observing reduced *Klebsiella* abundance (*p* = 0.01), increased *B. breve* abundance (*p* = 0.002), and increased d-3-phenyllactic acid levels (*p* < 0.001), with reduced RTI incidence and antibiotic use. In children with cystic fibrosis, Ray et al. (2022) [11] reported a 45% reduction in pulmonary exacerbations (incidence rate ratio [IRR] = 0.55, *p* = 0.01) and a 20% improvement in forced expiratory volume in one second (FEV1) (*p* = 0.001) with *Lacticaseibacillus rhamnosus* GG (LGG). Tabatabaii et al. (2024) [12] showed *L. reuteri* reduced *P. aeruginosa* colonization (1 vs. 7, *p* = 0.03) but without spirometric gains. Piloquet et al. (2024) [19] found that a synbiotic formula reduced LRTI risk by 23% (IRR = 0.77, *p* = 0.049), enhanced secretory immunoglobulin A (IgA), and lowered fecal pH. Mageswary et al. (2022) [16] reported beneficial effects of *B. lactis* Probio-M8 on acute respiratory tract infections, together with modulation of immune and gut microbial parameters.

### 3.6. Preclinical Evidence of Gut–Lung Axis in Neonatal Lung Diseases

Preclinical studies have elucidated mechanistic links between gut microbiota perturbations, inflammatory signaling, and neonatal lung disease, providing causal insights beyond clinical associations. Shen et al. (2024) [31] demonstrated that *L. plantarum* L168 improved alveolarization, with increased radial alveolar counts and reduced mean linear intercepts, reduced pulmonary IL-6 and TNF-α levels, and restored the expression of the tight-junction proteins occludin and claudin-1 in BPD pups. Fan et al. (2022) [22] observed hyperoxia-induced gut dysbiosis (increased *Proteobacteria* and decreased *Bacteroidetes*), associated with lung neutrophilia (*p* = 0.021). Abdelgawad et al. (2023) [25] found that hyperoxia downregulated antimicrobial peptides (AMPs), including lysozyme, altered gut microbial composition (increased *Staphylococcus* abundance), and lysozyme administration-reversed lung pathology. Albarracin et al. (2024) [23] showed that *L. rhamnosus* CRL1505 or *L. plantarum* MPL16 improved survival in pneumococcal pneumonia (60% vs. 0%) and modulated TNF-α and IFN-γ expression. Felix et al. (2018) [27] demonstrated that segmented filamentous bacteria (SFB) reduced CD47, enhanced neutrophil phagocytosis, and decreased pulmonary bacterial load (70-fold, *p* < 0.001). Overall, these preclinical findings provide mechanistic evidence supporting the gut–lung axis as a key modulator of neonatal lung injury, highlighting the therapeutic potential of microbiota-targeted interventions.

### 3.7. Integrated Omics and Clinical Correlates in Cystic Fibrosis

Integrative omics approaches provide high-resolution insights into the gut–lung axis and its potential role in early cystic fibrosis (CF) pathophysiology. Frayman et al. (2024) [20] conducted a prospective study in 39 infants with CF, integrating stool metagenomics, bronchoalveolar lavage fluid 16S rRNA sequencing, and plasma metabolomics. Airway *Veillonella* abundance was associated with greater airway α-diversity and lower inflammatory profiles, whereas inverse associations were observed with *Pseudomonas*. Stool *Veillonella* abundance was also associated with airway microbial diversity, while circulating metabolites, including uridine and cytidine, correlated with airway inflammatory markers. These findings support the potential use of stool-derived microbial and circulating metabolic markers as indirect indicators of lower-airway ecology and inflammation. However, they do not establish that fecal microbiota profiles are direct surrogates for lower-airway microbiota.

### 3.8. Mechanistic Insights into the Gut–Lung Axis: The “More Gut in the Lung” Phenomenon and ACE2

The gut–lung axis represents a complex and bidirectional network integrating intestinal and pulmonary homeostasis through coordinated microbial, epithelial, and immunological interactions. Under physiological conditions, the lung harbors a low-biomass but functionally relevant microbiota, primarily shaped by microbial immigration from the upper airways and, indirectly, from the gastrointestinal tract [39]. However, in acute pathological states such as sepsis, trauma, or acute respiratory distress syndrome, this equilibrium is disrupted, leading to the phenomenon defined as “more gut in the lung”, characterized by an increased presence of enteric-derived bacteria, including *Bacteroidetes* and *Enterobacteriaceae*, within the pulmonary microbiota [39,40]. This shift is driven by increased intestinal permeability (“leaky gut”) in parallel with alveolar–capillary barrier dysfunction, enabling microbial translocation and amplifying pulmonary inflammation and tissue injury [39,40]. Notably, intestinal dysbiosis may precede and contribute to pulmonary microbial alterations, supporting a systemic model in which gut-derived signals actively shape lung immunity and disease progression [39,41,42]. Within this integrated framework, angiotensin-converting enzyme 2 (ACE2) acts as a key molecular interface linking microbiota composition to host susceptibility. Highly expressed in both alveolar type II pneumocytes and intestinal enterocytes, ACE2 regulates amino acid transport, antimicrobial peptide production, and gut microbial ecology, while simultaneously serving as the entry receptor for SARS-CoV-2 [42]. Importantly, dysbiotic and pro-inflammatory gut environments are associated with increased ACE2 expression, creating a permissive niche for viral infection and facilitating systemic dissemination from the intestine to distant organs [42]. Moreover, ACE2 contributes to the modulation of the renin–angiotensin system and inflammatory signaling, linking microbial imbalance to both epithelial barrier disruption and systemic immune dysregulation [42]. Collectively, the convergence of gut dysbiosis, barrier dysfunction, microbial translocation, and ACE2-mediated pathways delineates a unified pathophysiological framework underlying the “more gut in the lung” phenomenon and its role in determining the severity of respiratory diseases, including COVID-19 [39,40,41,42].

## 4. Discussion

This systematic review identified a focused body of clinical and preclinical evidence supporting the biological plausibility and potential clinical relevance of the gut–lung axis during early life. Across the included studies, three recurring themes emerged: the coordinated development and subsequent divergence of microbial communities across mucosal sites; associations between intestinal microbial or metabolic alterations and respiratory phenotypes; and the potential modulation of pulmonary immunity through microbial metabolites, epithelial-barrier signaling, and systemic immune pathways. Nevertheless, the available evidence remains heterogeneous, and the respective contributions of microbial composition, microbial function, host susceptibility, environmental exposures, disease severity, and treatment-related effects cannot yet be clearly separated. The pediatric gut–lung axis should therefore be interpreted as a developmental and mechanistic framework rather than an established target for routine clinical intervention.

Evidence from longitudinal neonatal cohorts indicates that gut, oral, and upper-airway microbial communities initially share a substantial proportion of taxa but progressively develop niche-specific configurations during infancy [13,17,18]. Reyman et al. identified cross-site microbial networks associated with susceptibility to respiratory infections, whereas Wang et al. and Yang et al. documented early gut–pharyngeal overlap followed by divergence in term and preterm neonates [13,17,18]. Beneficial early colonizers, including *Dolosigranulum* and *Bifidobacterium*, may contribute to the stability of these microbial networks, while their depletion has been associated with increased susceptibility to respiratory tract infections [18]. However, correlated microbial trajectories do not establish direct microbial transfer between anatomical compartments. Stool and airway samples represent distinct ecological niches, and fecal microbiota profiles cannot currently be considered validated surrogates for lower-airway ecology.

The timing of microbial exposure may be as important as the presence or absence of individual taxa. Early life has been conceptualized as both a window of susceptibility, during which microbial disruption may exert persistent effects on immune and epithelial development, and a window of opportunity, during which appropriately timed environmental, nutritional, or microbial exposures may promote immune tolerance and mucosal homeostasis [30,43]. The duration of this window in humans remains uncertain and is unlikely to be identical across microbial, epithelial, metabolic, and immune developmental processes. This temporal framework is consistent with McDade’s ecological model of inflammation, which proposes that microbial environments encountered during early development contribute to the calibration of inflammatory regulation and may influence subsequent trajectories of chronic inflammatory disease [32]. Applied to pediatric respiratory health, this model suggests that early microbial deprivation or disruption could affect later respiratory susceptibility through persistent changes in immune regulation rather than through the persistence of a single dysbiotic signature. This interpretation remains conceptual and does not independently establish a causal gut–lung pathway.

The hygiene hypothesis and its subsequent refinements provide an additional framework for interpreting the relationship between environmental microbial exposure, immune maturation, and asthma. Contemporary formulations emphasize exposure to diverse environmental microorganisms and evolutionarily established microbial partners rather than insufficient personal hygiene itself [33,44]. Farm-associated microbial exposure represents one of the most consistent epidemiological examples. Depner et al. showed that maturation of the gut microbiome during the first year of life partly mediated the protective association between farm exposure and childhood asthma [15]. This observation connects environmental microbial diversity, microbial metabolic capacity, and respiratory immune development. Nevertheless, differences in diet, animal contact, household characteristics, air quality, and lifestyle may also contribute to the observed association. Delivery mode, infant feeding, antibiotic exposure, diet, and environmental microbial diversity should therefore be considered interacting determinants within a broader developmental network rather than isolated causal exposures.

The included experimental studies support a potential connection between intestinal microbial disruption and abnormal pulmonary development. Maternal or perinatal antibiotic exposure, postnatal growth restriction, and hyperoxia altered intestinal microbial communities, epithelial-barrier integrity, antimicrobial-peptide expression, and systemic inflammatory signaling in models of neonatal lung injury [24,25,28,34]. Reported mechanisms included increased intestinal permeability, circulating lipopolysaccharide, and activation of TLR4/MyD88/NF-κB signaling, accompanied by alveolar simplification, vascular abnormalities, pulmonary inflammation, or pulmonary hypertension. Complementary evidence published after completion of the predefined systematic search suggests that disruption of the gut microbiota–bile acid axis may precede severe bronchopulmonary dysplasia in preterm infants [45]. However, temporal precedence alone does not establish causality, because prematurity, oxygen exposure, enteral feeding, antibiotics, sepsis, and other intensive-care interventions may simultaneously influence intestinal microbial development and lung injury.

The effects of early antibiotic exposure are not uniform. Although several experimental studies suggest that maternal or neonatal antibiotics can alter microbial maturation and amplify pulmonary immune dysfunction [24,28], prolonged early-life antibiotic exposure altered the gut microbiota without exacerbating lung injury in a rat-pup model [46]. Oral vancomycin has also been shown to modify the murine lung microbiota and pulmonary immune responses [47]. These divergent findings indicate that respiratory consequences may depend on the antimicrobial agent, timing and duration of exposure, developmental stage, baseline microbiota, and type of pulmonary insult. Antibiotic-associated dysbiosis should therefore not be interpreted as producing a uniform respiratory phenotype. Similarly, the epidemiological association between childhood constipation and subsequent asthma reported by Huang et al. [14] represents an indirect clinical signal rather than direct evidence of dysbiosis, because microbiota composition, microbial metabolites, and intestinal inflammatory markers were not measured. Residual confounding, differences in healthcare utilization, and surveillance bias also remain possible.

Short-chain fatty acids (SCFAs) are among the most biologically plausible mediators of gut–lung communication. The included studies linked butyrate, propionate, pentanoate, and other microbial metabolites to G-protein-coupled receptor signaling, histone deacetylase inhibition, regulatory immune responses, and modulation of pulmonary inflammation [15,21,26,29]. Farm-exposed children showed greater microbiome maturation and increased production of microbial metabolites associated with a lower prevalence of asthma [15], while experimental studies demonstrated that SCFA supplementation or microbiota manipulation could modify allergic or inflammatory pulmonary phenotypes [21,26,29]. Complementary murine studies showed that butyrate and propionate promote peripheral or colonic Foxp3-positive regulatory T-cell differentiation through mechanisms involving histone deacetylase inhibition and increased acetylation at regulatory loci [48,49,50]. In human cells, butyrate and propionate enhanced the differentiation and suppressive function of induced regulatory T cells derived from adult peripheral blood and neonatal cord blood [51]. SCFAs may also act directly on structural cells: butyrate and propionate partially restored cytokine- and allergen-induced impairment of human bronchial epithelial-barrier function [52]. These findings support several potential pathways connecting intestinal microbial metabolism with pulmonary immune regulation and airway integrity. However, much of this evidence derives from in vitro exposure or experimental supplementation, and the concentrations reaching pulmonary tissues under physiological conditions in infants and children remain insufficiently characterized.

Microbial metabolites should not be considered in isolation. Human milk contains oligosaccharides, polyphenols, microorganisms, and other immunometabolically active components that can shape the developing intestinal ecosystem. Human milk oligosaccharides act as selective substrates for infant-associated microorganisms, whereas polyphenols and their microbial metabolites may influence microbial composition, metabolic activity, immune maturation, and epigenetic regulation [30,53]. Interactions among human milk oligosaccharides, polyphenols, the microbiota, and SCFA production provide a biologically plausible link between breastfeeding and immune development. Nevertheless, direct evidence connecting these interactions with pediatric respiratory outcomes remains limited. Human milk polyphenol–microbiota interactions should therefore be presented as a promising mechanistic hypothesis rather than an established pathway of respiratory protection.

The relationship between intestinal microbial development and childhood asthma is supported by converging epidemiological and experimental observations, but no universal asthma-associated microbial signature has emerged. Differences across studies may reflect age at sampling, geography, diet, antibiotic exposure, sequencing methodology, asthma phenotype, treatment, and host genetics. In a multicenter cross-sectional study of children with a history of bronchiolitis, stool shotgun metagenomics identified associations between individual microbial species, asthma prevalence, and lung-function indices at six years of age; some associations differed according to host polygenic risk, suggesting that host genetics may modify microbiome–respiratory relationships [54]. A pilot study also reported differences in intestinal microbial profiles among children with mild or intermittent asthma [55]. Because these studies were cross-sectional, they cannot determine whether microbial differences preceded asthma, resulted from the disease, or reflected treatment, diet, or other environmental factors.

Additional preclinical evidence indicates that environmental microorganisms may influence allergic airway inflammation through immune and epigenetic mechanisms. Intranasal administration of *Acinetobacter lwoffii* protected against experimental asthma through IL-6-dependent induction of IL-10 and epigenetic changes in CD4-positive T cells, accompanied by alterations in the intestinal microbiota [56]. *Clostridium leptum* also attenuated allergic airway inflammation through activation of a tolerogenic dendritic-cell–regulatory T-cell axis in a murine model of dysbiosis-associated asthma [57]. These studies are mechanistically relevant but should not be directly extrapolated to microbiota-based prevention or treatment in children. Collectively, these findings support the involvement of microbial, regulatory T-cell, cytokine, and epigenetic mechanisms in the modulation of type 2 inflammation.

Recent pediatric studies broaden the clinical spectrum of the gut–lung axis beyond asthma. A birth cohort using metagenomic profiling identified associations between first-week gut microbial characteristics and severe viral lower respiratory tract infections during the first two years of life [58]. Integrated analysis of intestinal and lower-airway microbiota with metabolomic data identified cross-compartment relationships in children with pulmonary arterial hypertension associated with congenital heart disease [59]. Although the latter study included a small cross-sectional sample, it illustrates the potential value of combining microbial and metabolic data across anatomical compartments. These findings extend the clinical framework but remain primarily associative.

The gut–lung axis is likely bidirectional. Respiratory viral infections may alter intestinal microbial and metabolic homeostasis, while gut-derived immune and microbial signals may, in turn, influence pulmonary inflammation [60]. Complementary preclinical evidence supports this bidirectional crosstalk across different experimental settings, including microbiota-dependent immune-cell trafficking in pneumonia [61], probiotic-associated modulation of the pulmonary microbiome in neonatal calves [62], and coordinated intestinal and pulmonary responses during influenza A infection [63]. However, these heterogeneous animal models provide mechanistic context and should not be interpreted as direct evidence of clinical efficacy in children. Respiratory infection, pulmonary inflammation, altered nutrition, medications, and hospitalization may themselves modify intestinal microbial and metabolic homeostasis. In a murine model, respiratory syncytial virus infection altered gut microbial composition and sphingolipid metabolism, while inhibition of sphingosine kinase or sphingosine-1-phosphate signaling reduced pulmonary inflammation [64]. Thus, intestinal dysbiosis may sometimes be a downstream consequence of respiratory disease or its treatment rather than the initiating event. Establishing temporal directionality will require longitudinal sampling before, during, and after respiratory illness. Microbiota-transfer experiments provide additional support for potential causality while illustrating the complexity of community-level effects. Transfer of distinct human fecal microbial communities to germ-free mice produced different degrees of inflammation and impaired lung function in the offspring [65]. However, the use of germ-free recipients, differences among donor communities, and interspecies translation limit direct application to human infants.

The included randomized controlled trials indicate that probiotic and synbiotic interventions may influence respiratory infections, microbial composition, and selected immune outcomes [10,11,12,16,19]. However, the evidence does not demonstrate a consistent class effect. Interventions differed in microbial strain, dose, formulation, duration, age at administration, comparator, adherence assessment, and clinical endpoint. Some respiratory outcomes were secondary endpoints, and microbiota modification was not uniformly demonstrated. Sjödin et al. reported microbial and metabolic differences following synbiotic feeding [10], while Piloquet et al. observed a modest reduction in lower respiratory tract infections with a synbiotic infant formula [19]. In cystic fibrosis, studies of *Lacticaseibacillus rhamnosus* GG and *Limosilactobacillus reuteri* reported potential benefits in exacerbations, selected lung-function measures, or *Pseudomonas aeruginosa* colonization [11,12]. Probio-M8 was also associated with improvements in acute respiratory outcomes and selected immune or microbial parameters [16]. These findings are promising but remain strain-specific and should not be generalized to probiotics as a single therapeutic category. A trial of soluble corn fiber supplementation in children with asthma further illustrates the potential of dietary substrates to influence endogenous microbial metabolism [66]; interpretation nevertheless requires demonstration of reproducible target engagement and clinically meaningful respiratory benefit.

Preclinical interventions similarly demonstrate biological responsiveness rather than clinical efficacy. *Lactiplantibacillus plantarum* L168 improved alveolar morphology, inflammatory markers, and intestinal barrier-related outcomes in a hyperoxia model [31], while other orally administered lactobacilli modified alveolar macrophage responses and survival following respiratory infection [23]. Cystic fibrosis provides a useful model in which diet, antibiotics, intestinal dysfunction, airway infection, and inflammation interact. Price et al. showed that intestinal *Bacteroides*-associated propionate production modified systemic and pulmonary inflammatory parameters in a mouse model [21]. Frayman et al. integrated stool metagenomics, lower-airway microbiota profiling, and plasma metabolomics in infants with cystic fibrosis [20]. Associations between stool and airway taxa and between circulating metabolites and airway inflammatory markers support coordinated cross-compartment signaling, but they do not establish that stool microbial profiles are direct surrogates for lower-airway microbiota.

**Figure 2:**
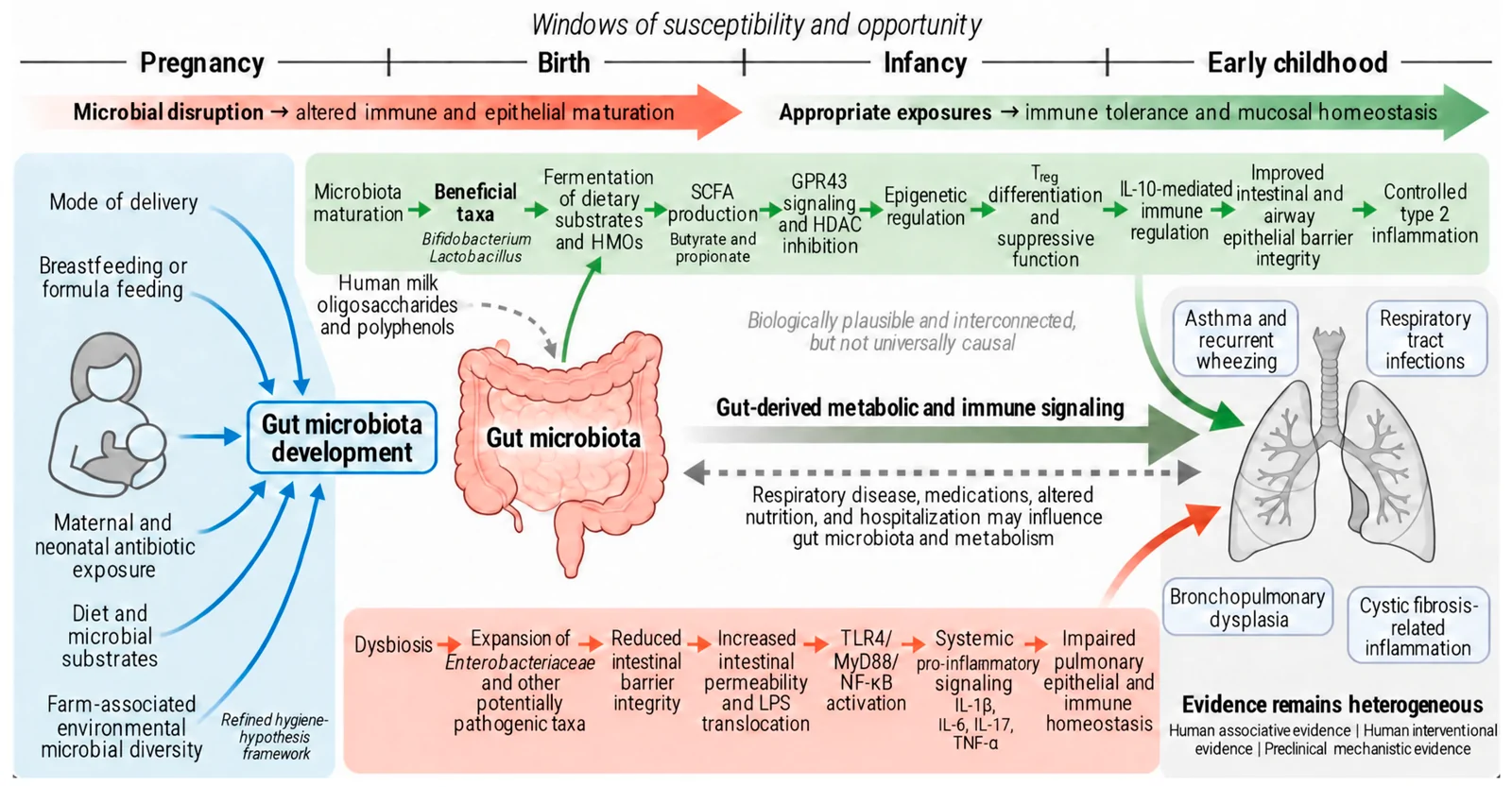
Developmental and mechanistic framework of the pediatric gut–lung axis.

Overall, current evidence supports the gut–lung axis as a plausible developmental framework involving microbial, metabolic, epithelial, and immune interactions. It does not yet justify routine microbiota-based diagnostics or interventions in neonatal and pediatric respiratory care. Longitudinal studies with repeated sampling before, during, and after respiratory disease are needed to establish directionality. Future investigations should integrate metagenomics, metabolomics, environmental exposure assessment, host genetics, epigenetic profiling, immune phenotyping, and standardized respiratory outcomes. Intervention trials should demonstrate target engagement by confirming the intended change in microbial composition or function and should include sufficiently long follow-up to determine whether biological changes translate into clinically meaningful and durable respiratory benefits. A mechanistic overview of the principal early-life exposures, signaling pathways, and respiratory outcomes is provided in Figure 2.

Early-life exposures may influence gut microbiota development during windows of susceptibility and opportunity. The upper pathway summarizes microbiota-derived metabolic and immunoregulatory mechanisms potentially supporting mucosal homeostasis, whereas the lower pathway depicts dysbiosis-associated inflammatory mechanisms potentially contributing to pediatric respiratory outcomes. The gut–lung relationship is bidirectional, biologically plausible, and interconnected, although the available evidence remains heterogeneous and does not establish universal causality. SCFA, short-chain fatty acid; HMO, human milk oligosaccharide; GPR43, G-protein-coupled receptor 43; HDAC, histone deacetylase; Treg, regulatory T cell; IL, interleukin; LPS, lipopolysaccharide; TLR4, Toll-like receptor 4; MyD88, myeloid differentiation primary response 88; NF-κB, nuclear factor kappa B; TNF-α, tumor necrosis factor alpha.

### Limitations

The principal limitation of this review concerns the sensitivity of the predefined systematic search strategy. Requiring the simultaneous presence of multiple concept blocks increased specificity but may have excluded relevant studies addressing only some dimensions of the pediatric gut–lung axis. Therefore, the studies included in the formal systematic synthesis may not capture the full breadth of the literature in this field. To mitigate this limitation, we performed a supplementary targeted PubMed search and citation tracking focused on environmental microbial exposures, developmental windows, microbial metabolites, regulatory T-cell differentiation, airway epithelial responses, epigenetic mechanisms, and human-milk bioactive compounds. Evidence identified through this supplementary approach was used exclusively to broaden the contextual and mechanistic interpretation and was not retrospectively incorporated into the PRISMA flow diagram, systematic evidence tables, or formal risk-of-bias assessment. Accordingly, although the predefined systematic component remained methodologically distinct, the complementary evidence synthesis was narrative and may be susceptible to selection bias.

Additional limitations arise from the considerable methodological heterogeneity across the included studies. Variability in study design, sequencing platforms, microbial classification pipelines, and biological samples—including stool and bronchoalveolar lavage fluid—hinders direct comparisons and reduces the consistency of the available evidence. Differences in probiotic strains, dosages, treatment durations, and intervention protocols further limit comparability. The relatively short follow-up of most randomized controlled trials restricts the assessment of long-term respiratory and immunological outcomes, while the limited stratification by microbial or immune biomarkers constrains mechanistic interpretation and the identification of differential responses among subgroups. Important evidence gaps also remain, including the paucity of longitudinal studies investigating maternal–infant microbial continuity, host–microbiota genetic interactions, and the timing of microbial exposures in relation to immune maturation. These limitations hinder the development of a coherent developmental framework and the translation of current evidence into precision microbiota-based interventions in pediatric respiratory medicine.

## 5. Conclusions

The gut–lung axis is emerging as a potentially important regulator of respiratory health in early life, integrating microbial, metabolic, and immunological signals across distant mucosal compartments. This systematic review identifies converging clinical and preclinical evidence suggesting that early microbial colonization, dysbiosis, and microbiota-derived metabolites such as short-chain fatty acids may influence systemic immune regulation and respiratory outcomes through multiple biological pathways. Probiotic, synbiotic, and metabolite-based interventions show promising potential; however, their clinical effects remain heterogeneous and require confirmation in larger, well-designed longitudinal and interventional studies. Multidimensional omics approaches may further improve the characterization of gut–lung interactions and facilitate the identification of noninvasive biomarkers and microbiota-related endotypes. Overall, the pediatric gut–lung axis represents a promising framework for respiratory immunology, but further mechanistic and clinical validation is required before microbiota-targeted strategies can be incorporated into routine pediatric respiratory care.

## Figures and Tables

**Table 1:** Clinical studies investigating the gut–lung axis in early life.

Author	Study Type	Population	Microbiota Focus	Respiratory Outcomes
Sjödin et al., 2023 [10]	RCT	Full-term infants	*L. paracasei* F19 + prebiotics	LRTI, immune modulation
Ray et al., 2022 [11]	RCT	CF children	*L. rhamnosus* GG	Exacerbations, FEV1
Tabatabaii et al., 2024 [12]	RCT	CF children	*L. reuteri*	Lung function, infections
Yang et al., 2021 [13]	Clinical	Preterm neonates	Gut-pharyngeal microbiota	Microbial correlation
Huang et al., 2021 [14]	Cohort	Children ≤ 18	Dysbiosis	Asthma incidence
Depner et al., 2020 [15]	Cohort	Children	SCFAs	Asthma prevalence
Mageswary et al., 2022 [16]	RCT	Infants/children	*B. lactis* Probio-M8	RTI symptoms
Wang et al., 2023 [17]	Observational	Neonates	Gut/pharyngeal microbiota	Composition
Reyman et al., 2021 [18]	Observational	Infants	Cross-niche microbiota	RTIs
Piloquet et al., 2024 [19]	RCT	Infants	Synbiotic formula	LRTI
Frayman et al., 2024 [20]	Longitudinal	CF infants	Multi-omics	Inflammation

**Table 2:** Preclinical (animal) studies investigating mechanistic aspects of the gut–lung axis.

Author	Study Type	Model	Microbiota Focus	Respiratory Outcomes
Price et al., 2024 [21]	Animal	CF mice	*Bacteroides*	Inflammation
Fan et al., 2022 [22]	Experimental	Neonatal mice	Dysbiosis	Lung morphology
Albarracin et al., 2024 [23]	Experimental	Infant mice	*L. rhamnosus*/*L. plantarum*	Survival
Willis et al., 2020 [24]	Experimental	Neonatal mice	Antibiotics	BPD
Abdelgawad et al., 2023 [25]	Experimental	Neonatal mice	AMPs	Inflammation
Papotto et al., 2023 [26]	Experimental	Neonatal mice	SCFAs	Type 2 immunity
Felix et al., 2018 [27]	Experimental	Rag−/− mice	SFB	Pneumonia severity
Alhasan et al., 2023 [28]	Experimental	Mouse offspring	Dysbiosis	Asthma
Liu et al., 2020 [29]	Experimental	BALB/c mice	Butyrate	Inflammation
Zhuang et al., 2024 [30]	Experimental	C57BL/6 mice	Gut-lung axis	Pneumonia
Shen et al., 2024 [31]	Preclinical	Neonatal rats	*L. plantarum*	BPD
McDaneld et al., 2024 [32]	Preclinical	Calves	Probiotics	Microbiota
Hao et al., 2023 [33]	Preclinical	BALB/c mice	TCM	Inflammation
Wedgwood et al., 2020 [34]	Experimental	Neonatal Sprague–Dawley rats	Gut microbiota dysbiosis; *Lactobacillus reuteri* DSM 17938	Pulmonary hypertension; right ventricular hypertrophy

## Data Availability

No new data were created or analyzed in this study. Data sharing is not applicable to this article.

## Supplementary Materials

The following supporting information can be downloaded at https://www.mdpi.com/article/10.3390/nu18193260/s1, Supplementary File S1: Full database-specific search strategies for PubMed, Web of Science, and Scopus; Table S1: Summary of reviews retained for complementary contextual evidence; Table S2: Newcastle–Ottawa Scale assessment of observational studies; Table S3: SYRCLE risk-of-bias assessment of preclinical studies; Table S4: RoB 2 assessment of randomized controlled trials; Checklist S1: PRISMA 2020 checklist.

## Abbreviations

ACE2	Angiotensin-converting enzyme 2
aHR	Adjusted hazard ratio
AMP	Antimicrobial peptide
BALF	Bronchoalveolar lavage fluid
BPD	Bronchopulmonary dysplasia
CF	Cystic fibrosis
CI	Confidence interval
COVID-19	Coronavirus disease 2019
FEV1	Forced expiratory volume in one second
FITC	Fluorescein isothiocyanate
Foxp3	Forkhead box P3
G-CSF	Granulocyte colony-stimulating factor
GPR43	G protein-coupled receptor 43
HDAC	Histone deacetylase
HMO	Human milk oligosaccharide
IFN-γ	Interferon gamma
IgA	Immunoglobulin A
IL	Interleukin
ILC2	Group 2 innate lymphoid cell
IRR	Incidence rate ratio
LGG	Lacticaseibacillus rhamnosus GG
LPS	Lipopolysaccharide
LRTI	Lower respiratory tract infection
MeSH	Medical Subject Headings
MIP-1β	Macrophage inflammatory protein-1 beta
MyD88	Myeloid differentiation primary response 88
NEC	Necrotizing enterocolitis
NF-κB	Nuclear factor kappa B
NOS	Newcastle–Ottawa Scale
OR	Odds ratio
OTU	Operational taxonomic unit
PICOS	Population, intervention or exposure, comparator, outcomes, and study design
PRISMA	Preferred Reporting Items for Systematic Reviews and Meta-Analyses
PROSPERO	International Prospective Register of Systematic Reviews
RCT	Randomized controlled trial
RoB 2	Cochrane Risk of Bias 2 tool
RORγt	Retinoic acid receptor-related orphan receptor gamma t
rRNA	Ribosomal ribonucleic acid
RSV	Respiratory syncytial virus
RTI	Respiratory tract infection
S1P	Sphingosine-1-phosphate
SARS-CoV-2	Severe acute respiratory syndrome coronavirus 2
SCFA	Short-chain fatty acid
SFB	Segmented filamentous bacteria
SPHK	Sphingosine kinase
SYRCLE	Systematic Review Centre for Laboratory Animal Experimentation
TCM	Traditional Chinese medicine
TGF-β	Transforming growth factor beta
Th17	T-helper 17
TLR	Toll-like receptor
TLR4	Toll-like receptor 4
TNF-α	Tumor necrosis factor alpha
Treg	Regulatory T cell
