# Supplementary material for: Early-Life Gut Microbiota and the Pediatric Gut–Lung Axis: A Systematic Review of Clinical, Preclinical, and Mechanistic Evidence
Source: Nutrients. 2026 Oct 2;18(19):3260. doi: 10.3390/nu18193260 (PMC13666610; doi:10.3390/nu18193260)
Supplement: Supplementary file 1 [file nutrients-18-03260-s001.zip › Checklist S1.pdf]

PRISMA 2020 Checklist

This checklist has been completed based on the submitted manuscript “Early-Life Gut Microbiota and the Pediatric Gut–Lung Axis: A Systematic Review of Clinical, Preclinical, and Mechanistic Evidence”. Page numbers refer to the location in the manuscript where each item is addressed.

Section/topic	Item	Checklist item	Reported on page #
Title	1	Identify the report as a systematic review.	Title page
Abstract	2	Provide a structured summary including background, objectives, data sources, eligibility criteria, study appraisal, synthesis, results, and conclusions.	Abstract
Introduction	3	Describe the rationale for the review in the context of what is already known.	p. 3
Objectives	4	Provide an explicit statement of the objective(s).	p. 3
Methods	5	Specify the inclusion and exclusion criteria for the review and how studies were grouped for the syntheses.	p. 4–5
Methods	6	Describe all information sources in the search	p. 4

		(databases, registers, websites, etc.), date last searched.	
Methods	7	Present the full search strategies for all databases, including any filters and limits used.	Supplementary Material
Methods	8	Specify the methods used to assess risk of bias in the included studies.	p. 5
Results	16	Provide results of the search and selection process, ideally using a flow diagram.	p. 6 + Figure 1 (PRISMA flow diagram)
Results	17	Cite studies that met many but not all inclusion criteria (if applicable).	p. 6
Discussion	23	Provide a general interpretation of the results in the context of other evidence, and implications for future research, practice, or policy.	p. 12–14
Funding	27	Describe sources of funding for the review and other support; role of funders.	Declarations
