# Supplementary material for: Early-Life Gut Microbiota and the Pediatric Gut–Lung Axis: A Systematic Review of Clinical, Preclinical, and Mechanistic Evidence
Source: Nutrients. 2026 Oct 2;18(19):3260. doi: 10.3390/nu18193260 (PMC13666610; doi:10.3390/nu18193260)
Supplement: Supplementary file 1 [file nutrients-18-03260-s001.zip › Supplementary File S1.pdf]

Supplementary Material S1 - Full database-specific search strategies for PubMed, Web of Science, and Scopus.

Databases searched

- PubMed
- Web of Science
- Scopus

Search period

- January 1, 2014 – March 31, 2025
- Last search performed: March 31, 2025

Filters applied

- Peer-reviewed articles
- English language
- Human and relevant preclinical studies
- Age: 0–16 years

("Gut Microbiota" OR "Intestinal Microbiome" OR "Infant Microbiota")
AND ("Lung" OR "Pulmonary" OR "Respiratory Tract" OR "Airway Microbiome")
AND ("Infant" OR "Child" OR "Pediatric" OR "Neonatal")
AND ("Immune Development" OR "Cytokines" OR "Inflammation" OR "Toll-like Receptors")
AND ("Breastfeeding" OR "Cesarean Section" OR "Antibiotics")
AND ("Asthma" OR "Bronchiolitis" OR "Wheezing" OR "Respiratory Infection")
AND ("Probiotics" OR "Prebiotics" OR "Microbiota Modulation")

This single search string was developed in PubMed and applied to Web of Science and Scopus with minor syntax adjustments (e.g., field tags, quotation marks, truncation symbols), without conceptual modifications.

Database-specific notes

PubMed

- MeSH terms used where available (e.g., “Gut Microbiota”[MeSH], “Lung”[MeSH], “Infant”[MeSH]).
- Free text combined with MeSH using OR.
- Boolean operators (AND/OR) applied as shown.
- Filters: English, publication date 2014–2025.

Web of Science

- Same conceptual search string used.
- Field: *Topic* (TS).
- No MeSH terms.
- Minor syntax adjustments (e.g., field tags, truncation).
- Filters: English, publication date 2014–2025.

Scopus

- Same conceptual search string used.
- Field: *TITLE-ABS-KEY*.
- Adjusted quotation marks and truncation symbols as required.
- Filters: English, publication date 2014–2025.

Note:

- The same conceptual query was used for all databases to maximize sensitivity and ensure consistency in retrieval.
- The exact search date and filters are reported above to support reproducibility.
