# Supplementary material for: Early-Life Gut Microbiota and the Pediatric Gut–Lung Axis: A Systematic Review of Clinical, Preclinical, and Mechanistic Evidence
Source: Nutrients. 2026 Oct 2;18(19):3260. doi: 10.3390/nu18193260 (PMC13666610; doi:10.3390/nu18193260)
Supplement: Supplementary file 1 [file nutrients-18-03260-s001.zip › Supplementary Table S1.pdf]

Supplementary Table S1 – Summary of reviews retained for complementary contextual evidence. This table supports the systematic review registered in PROSPERO (ID: CRD420251062905), which details the methodology and eligibility criteria used for study selection and assessment.

Author and Year	Review Type	Microbiota Focus	Respiratory Themes	Key Insights	Conceptual Relevance	Translational Relevance
Hufnagl et al., 2020 [1]	Narrative Review	Gut and Lung Microbiota Dysbiosis, Antibiotics, Probiotics, Environmental Factors	Asthma, Bronchial Hyperresponsiveness, Inflammation	Microbiota dysbiosis correlates with asthma prevalence; environmental exposures can modulate lung microbiota	Dysbiosis disrupts gut-lung axis leading to immune hypersensitivity and respiratory diseases	Relevant for asthma prevention; probiotic interventions require more research
Kloepfer & Kennedy, 2023 [2]	Narrative Review	Gut and lung microbiota, viral infections (RSV, Rhinovirus), antibiotic exposure	Asthma, Wheezing, Respiratory Infections, Airway Inflammation	Early viral infections alter microbiota and increase asthma risk; microbial diversity may protect against asthma	Disruptions in early-life microbiota and viral infections contribute to immune dysregulation and asthma development	Relevant for early-life asthma prevention; targeting microbiota could be a therapeutic strategy
Yuksel et al., 2023 [3]	Narrative Review	Gut and lung microbiota, probiotics (Lactobacillus, Bifidobacterium), antibiotic exposure	Asthma, Bronchopulmonary Dysplasia, COPD, Pneumonia, Cystic Fibrosis, Influenza, Lung Cancer, COVID-19	Lung microbiota composition influences respiratory disease severity; probiotics may offer therapeutic potential	Restoring microbiota balance could improve respiratory health through the gut-lung axis	Potential for probiotics as lung-targeted therapies; novel drug delivery approaches needed
Perdijk et al., (2024) [4]	Review.	Gut and lung microbiota, gut-lung axis,	Asthma, COPD, lung cancer, cystic fibrosis,	Microbiota-targeted therapies could reduce	SCFAs and probiotics offer potential for reducing inflammation	Highlights microbiota-targeted therapies for

		microbial metabolites (e.g., SCFAs)	immune homeostasis	lung disease risk. Dysbiosis exacerbates asthma, COPD, and cystic fibrosis.	and disease progression.	respiratory diseases.
Willis & Ambalavanan, 2020 [5]	Narrative Review	Gut and lung microbiota interactions in preterm infants with necrotizing enterocolitis (NEC) and bronchopulmonary dysplasia (BPD) Dysbiosis and microbial translocation from gut to lung Influence of Ureaplasma spp., Proteobacteria, and Firmicutes on NEC/BPD risk	Bronchopulmonary dysplasia (BPD) and lung inflammation in preterm infants Increased ventilation needs and oxygen requirement due to systemic inflammation	NEC induces systemic inflammation, leading to lung dysbiosis and BPD development Gut microbiome changes (Proteobacteria dominance) precede NEC onset Ureaplasma spp. colonization increases NEC and BPD risk Microbial metabolites (SCFAs) can regulate lung inflammation	Gut dysbiosis strongly influences neonatal lung outcomes Targeting gut microbiota (e.g., probiotics) may reduce NEC and BPD incidence Microbial translocation from gut to lung represents a key pathway for systemic inflammation	Supports early microbiota-targeted interventions (e.g., probiotics, reducing antibiotic overuse) Highlights need for longitudinal gut-lung microbiota studies in neonates
Yang et al., 2021 [6]	Narrative Review	Gut-lung axis and microbiota dysbiosis in BPD Impact of Proteobacteria, Firmicutes, Ureaplasma	Bronchopulmonary dysplasia (BPD) in preterm infants Lung inflammation and immune dysregulation	BPD is closely linked to gut dysbiosis and immune activation. Early antibiotic	Microbiota-targeted therapies (e.g., probiotics, fecal microbiota transplantation - FMT) may prevent BPD. Early	Supports FMT and probiotics as potential BPD therapies. Highlights importance of early-life

		spp., Escherichia, and Shigella on BPD progression<br>Influence of maternal antibiotic exposure (MAE) and postnatal antibiotics on gut microbiota development	due to gut microbiota alterations	use disrupts gut microbiota, prolongs pathogen colonization, and increases lung inflammation.<br>Hyperoxia alters gut-lung microbiota composition, exacerbating BPD.<br>SCFAs and probiotics could mitigate BPD by restoring gut microbiota balance.	microbiota monitoring in preterm infants could identify high-risk BPD cases.	microbiota interventions.
Ranucci et al., 2017 [7]	Narrative Review	Early-life gut microbiota composition and lung health<br>Impact of delivery mode, feeding type, antibiotics, and infections on microbiota development<br>Role of gut microbiota in asthma, cystic	Asthma risk due to reduced microbial diversity in the first year of life<br>Cystic fibrosis (CF): Gut dysbiosis contributes to systemic inflammation and lung disease progression<br>Bronchopulmonary dysplasia (BPD) and its potential	Early-life microbiota composition influences lung disease risk<br>Gut dysbiosis is linked to asthma, CF, and potentially BPD<br>Breastfeeding and early microbial exposure reduce respiratory	Microbiota-targeted therapies (e.g., probiotics, SCFA supplementation) may improve lung health<br>Early gut microbiota profiling could identify at-risk infants for asthma or CF progression	Supports early gut microbiota interventions to prevent chronic lung diseases<br>Suggests probiotics and dietary modifications for CF and asthma

		fibrosis (CF), and bronchopulmonary dysplasia (BPD)	link with poor nutrition and gut alterations	disease risk<br>Probiotics and prebiotics may help modify lung disease progression		
Kahhaleh et al., 2024 [8]	Narrative Review	Gut-lung axis and microbiota dysbiosis in early-life asthma development<br>Impact of delivery mode, feeding type, antibiotic exposure, and environmental factors on microbiota establishment<br>Role of Bifidobacterium, Faecalibacterium, Akkermansia, Clostridium, Lactobacillus, and Proteobacteria in asthma susceptibility	Asthma risk due to microbiota dysbiosis in infancy<br>Increased allergic sensitization linked to gut microbial alterations<br>Inflammatory pathways activation associated with early gut dysbiosis	Gut microbiota alterations in infancy strongly influence asthma risk<br>Early-life antibiotic use disrupts microbiota, increasing asthma susceptibility<br>Farm exposure and probiotic-enriched diets may reduce asthma incidence<br>Microbiota-targeted therapies (probiotics, SCFAs) show potential for asthma prevention	Early-life microbiota profiling could help predict asthma risk<br>Interventions targeting gut microbiota (e.g., prebiotics, probiotics) may prevent asthma onset	Supports microbiota-targeted strategies for asthma prevention<br>Highlights potential for prebiotics, probiotics, and diet-based interventions

Xue et al., 2023 [9]	Narrative Review	Gut dysbiosis and its impact on childhood asthma and bronchiolitis Influence of perinatal factors: delivery mode, antibiotic exposure, breastfeeding Interactions between gut and lung microbiota within the gut-lung axis (GLA)	Asthma: Gut microbiota alterations linked to airway hyperresponsiveness Bronchiolitis: Correlation between gut microbiota and respiratory syncytial virus (RSV) infection Pulmonary inflammation and immune dysregulation driven by gut microbial imbalance	Early-life gut microbiota composition strongly influences asthma and bronchiolitis risk Antibiotic use in infancy disrupts gut microbiota, increasing asthma susceptibility Early exposure to diverse microbiota (e.g., rural environments, breastfeeding) protects against respiratory diseases Microbiota-targeted interventions (probiotics, prebiotics, SCFAs) show potential in asthma prevention	Early gut microbiota profiling could help predict asthma risk Targeted interventions (e.g., probiotics, prebiotics, diet) may reduce susceptibility to respiratory diseases	Supports microbiota-targeted strategies for asthma prevention Highlights the potential of prebiotics, probiotics, and dietary interventions
Zhu et al., 2021 [10]	Narrative Review	Gut-lung axis and microbiota dysbiosis in pediatric respiratory infections (asthma,	Respiratory infections such as asthma, bronchiolitis, recurrent infections,	Gut microbiota composition strongly correlates with severity of pediatric	Modulating gut microbiota (e.g., SCFAs, probiotics) could improve pediatric respiratory health. Early	Highlights potential of probiotics and SCFAs as preventive/therapeutic tools for

		bronchiolitis, RSV, and tuberculosis)	and tuberculosis	respiratory infections. Alterations in Lachnospira, Veillonella, and SCFAs predispose children to asthma. Dysbiosis impacts immune homeostasis in diseases like RSV and tuberculosis.	microbial interventions may help prevent respiratory infections.	pediatric respiratory diseases.
Piersigilli et al., 2020 [11]	Narrative Review	Gut-lung axis in preterm lung disease, particularly RDS and BPD. Influence of nutrients (amino acids, lipids, vitamins) and microbiota on neonatal lung development and immune modulation. Impact of human milk, probiotics, and prebiotics on lung	Bronchopulmonary dysplasia (BPD)<br>- Respiratory distress syndrome (RDS)<br>- Lung inflammation and developmental impairments due to premature birth and nutritional factors	Human milk reduces BPD risk compared to formula feeding<br>- Early microbiota composition impacts lung development, with SCFAs and probiotics modulating immune responses<br>- Maternal nutrition, vitamin deficiencies, and antibiotics impact preterm lung outcomes	Microbiota modulation (e.g., breast milk, probiotics, DHA supplementation) may reduce lung disease risk in preterm infants<br>- Early-life microbial profiling could predict lung health outcomes	Supports microbiota-targeted strategies (prebiotics, probiotics) to prevent BPD in preterm infants<br>- Highlights the role of DHA, Vitamin A, and maternal milk in lung health

		health in preterm infants				
Zama et al., 2022 [12]	Narrative Review	Gut microbiota composition and its dysbiosis; specific metabolites such as short-chain fatty acids (SCFAs) and desaminotyrosine (DAT).	Respiratory tract infection, bronchiolitis severity, respiratory viral	Gut dysbiosis is associated with increased RTI susceptibility. Early microbiota shaping influences long-term respiratory health.	Probiotic and prebiotic interventions could potentially mitigate RTI risks via gut-lung axis modulation.	High potential for microbiota-targeted therapies in pediatric respiratory care.
Mohan Pammi et al., 2018 [13]	Systematic Review	Microbiota Factor Examined: Airway microbiome composition (16S rRNA sequencing). Bacterial diversity (alpha- and beta-diversity indices). Temporal changes in bacterial load. Role of specific bacterial taxa (e.g., Proteobacteria, Firmicutes, Lactobacilli).	Development and severity of Bronchopulmonary Dysplasia (BPD). Oxygen dependence at 36 weeks postmenstrual age (PMA). Inflammation and lung injury markers.	Airway microbiome composition plays a role in BPD pathogenesis. Specific bacterial profiles could serve as biomarkers for early identification of at-risk infants. Need for microbiome-targeted interventions (e.g., probiotics, microbiota optimization).	No direct correlation between gut microbiota and airway microbiome was evaluated in the included studies. Suggests maternal microbiome, delivery mode, and early nutrition could play a role in microbial colonization.	High clinical relevance for neonatal care and BPD prevention. Potential for microbiome-based therapies (e.g., probiotics, microbiota transplantation). Highlights the need for standardized microbiome analysis in neonates.
Stricker, S., Hain, T., Chao, C.-M.,	Narrative Review	Microbiota Factor Examined: Intestinal	Incidence and severity of bronchopulm	Microbiota composition is linked to	Gut microbiota modulation could serve as	High potential for using microbiota-

Rudloff, S., 2022 [14]		and respiratory microbiota composition (via 16S rRNA sequencing). Role of short-chain fatty acids (SCFAs) in immune regulation. Influence of gut microbiota on lung diseases (gut–lung axis). Impact of probiotics, prebiotics, and synbiotics on pediatric lung health.	onary dysplasia (BPD). Cystic fibrosis (CF) exacerbations and lung microbiome alterations. Association of asthma and atopy with gut microbiota composition. COVID-19 severity and respiratory microbiome changes.	respiratory disease severity. Probiotic and prebiotic interventions may modulate immune responses and prevent lung infections.	a therapeutic approach for pediatric lung diseases. Early-life microbiome shaping impacts long-term respiratory health.	based biomarkers in respiratory disease prediction. Potential role of probiotics and synbiotics in preventing respiratory infections, asthma, and CF exacerbations.
Tirone, C., Pezza, L., Paladini, A., et al., 2019 [15]	Narrative Review	Microbiota Factor Examined: Gut and lung microbiota composition (via 16S rRNA sequencing). Microbial dysbiosis and its immune-modulatory effects. Role of facultative anaerobes and short-chain fatty	Incidence and severity of NEC, BPD, and LOS in preterm infants. Growth impairment linked to microbiota composition.	Microbiota dysbiosis contributes to neonatal diseases, including NEC, BPD, sepsis, and growth impairment. SCFAs regulate immune homeostasis, and gut dysbiosis can impair immune function.	Targeting microbiota could prevent respiratory and gastrointestinal diseases in preterm infants. Probiotics, prebiotics, and maternal microbiota modulation may improve neonatal outcomes.	High potential for microbiota-based interventions in neonatal care. Possible role for probiotics, synbiotics, and targeted microbiota therapies in preventing NEC, BPD, and sepsis.

		acids (SCFAs).				
Gao et al., 2024 [16]	Narrative Review	Gut and lung microbiota, short-chain fatty acids (SCFAs), gut-lung axis	Bronchopulmonary dysplasia (BPD), lung inflammation, immune modulation	Microbiome and SCFAs are involved in BPD pathogenesis. Dysbiosis affects lung inflammation, barrier integrity, and immune response. Potential for microbiome-targeted therapies.	SCFAs and microbiome interact bidirectionally; gut-lung axis influences immune responses and lung health. Restoring microbiota balance may help prevent or treat BPD.	Potential for microbiota-based therapies (probiotics, prebiotics, SCFAs supplementation) to prevent or treat BPD.
Colombo et al.,2024 [17]	Scoping Review	Airway microbiome, gut-lung axis, BPD association, perinatal factors	Bronchopulmonary dysplasia (BPD), immune modulation, lung colonization	Airway microbiome is shaped by perinatal factors (delivery mode, antibiotics, chorioamnionitis). BPD infants exhibit distinct microbial patterns. The gut-lung axis plays a role in early immune regulation.	Strong association between gut and airway microbiota, with shared bacterial communities. Disruptions in gut microbiome impact airway microbiota composition and function.	Potential for microbiome-targeted interventions (probiotics, maternal microbiome modulation, synbiotics). Need for controlled trials to explore preventive strategies for BPD.
Campbell et al., 2024 [18]	Narrative Review	Lower airway microbiome, oral-lung	Bronchopulmonary dysplasia (BPD),	Lung microbiota differs by age,	Bidirectional interactions between gut and lung	Potential for microbiota-based interventions

		axis, gut-lung axis, chronic lung disease associations	asthma, cystic fibrosis (CF), chronic lung infections	disease state, and environmental exposures. Chronic lung disease patients have altered microbiota, often with increased oral taxa translocation. The gut-lung axis plays a significant role in immune regulation and disease pathogenesis.	microbiota impact immune responses. Dysbiosis in the gut influences lung microbiota, possibly contributing to chronic respiratory conditions.	(probiotics, prebiotics, synbiotics) in preventing lung disease. Multi-omics approaches could identify microbiome-based biomarkers for early diagnosis and treatment.
Chan et al., 2020 [19]	Systematic Review & Meta-Analysis	Synbiotic interventions (probiotic + prebiotic) for RTI prevention	Incidence of respiratory tract infections (RTIs), antibiotic use	Synbiotic supplementation reduced RTI risk, particularly in adults. No significant evidence of publication bias. Antibiotic misuse for viral RTIs remains a concern, and synbiotics may help reduce unnecessar	Strong gut-lung axis connection in RTI prevention. Synbiotics help balance gut microbiota, leading to improved immune responses and reduced RTI incidence.	Supports the use of synbiotic interventions as a nutritional strategy for RTI prevention. Potential to reduce antibiotic overuse and antimicrobial resistance globally.

				y antibiotic use.		
Meirlaen et al., 2021 [20]	Narrative Review	Probiotics, prebiotics, synbiotics, gut-lung axis	Asthma, allergic rhinitis, immune modulation	While animal studies show benefits, human trials remain inconclusive. Probiotics given prenatally and postnatally may reduce atopy risk but do not significantly prevent asthma or allergic rhinitis.	Dysbiosis in early life contributes to immune dysregulation, promoting allergic diseases. Probiotic interventions may modulate immune responses but require further targeted research.	Potential for microbiome-targeted interventions in allergic disease prevention, but standardized trials are needed.
Pirr et al., 2025 [21]	Narrative Review	Respiratory microbiome development, gut-lung axis, perinatal influences	Neonatal respiratory microbiome composition, immune modulation, respiratory infections, asthma, BPD	Neonatal respiratory microbiota is shaped by gestational age, delivery mode, diet, and postnatal exposures. Altered microbiota composition is linked to increased risk of respiratory infections, asthma, and BPD.	The gut microbiota plays a key role in lung health via systemic immune modulation. SCFAs and microbial metabolites influence pulmonary immunity and resistance to respiratory pathogens.	Potential for microbiota-targeted interventions to improve neonatal respiratory health, including probiotics, prebiotics, and maternal microbiome modulation.

Musso et al., 2020 [22]	Narrative Review	Gut, lung, and skin microbiome in allergic diseases	Asthma, bronchial hyperresponsiveness, allergic inflammation	Microbiota plays a key role in allergic disease pathogenesis. Early-life dysbiosis due to cesarean delivery, antibiotic exposure, or formula feeding increases allergic disease risk. Probiotics and prebiotics may have therapeutic potential but require further research.	The gut microbiome modulates lung immunity via SCFA production and immune signaling. Restoring microbial balance could be a novel approach for preventing allergic diseases.	Microbiome-based interventions (probiotics, prebiotics) could help modulate allergic disease risk in early life. Further controlled trials are needed to assess therapeutic efficacy.
Yang & Dong, 2020 [23]	Narrative Review	Lung microbiota dysbiosis, probiotics, gut-lung axis	Bronchopulmonary dysplasia (BPD), inflammation, immune modulation	Lung microbiota plays a key role in BPD pathogenesis. Probiotics such as Lactobacillus and Bifidobacterium could help restore microbial balance, reduce inflammation, and potentially improve	The gut-lung axis is central to BPD pathogenesis. Gut microbiota dysbiosis exacerbates lung inflammation, while probiotics may offer therapeutic benefits.	Probiotics may be a promising intervention for BPD prevention and management. Further clinical trials are needed to establish efficacy and safety in preterm infants.

				BPD outcomes.		
Sun et al., 2021 [24]	Narrative Review	Respiratory tract microecology, gut-lung axis, bacterial diversity, and microbial dysbiosis	Bronchopulmonary Dysplasia (BPD) and related inflammatory and immunological changes	BPD development is influenced by early-life microbiota colonization patterns; microbiome disruptions may predict BPD severity and	Dysbiosis in the gut-lung axis plays a significant role in BPD progression; interventions targeting microbiota balance could reduce lung inflammation and improve outcomes.	Microbiota-targeted interventions such as probiotics or antimicrobial strategies could reduce BPD incidence and severity.
Pulvirenti et al., 2019 [25]	Narrative Review	Lower airway microbiota, gut-lung axis, asthma, infections, immunity, probiotics	Asthma, airway inflammation, respiratory infections, immune modulation	Microbiota alterations are linked to respiratory diseases; probiotics show potential for improving lung immunity	Gut microbiota influences lung immunity and inflammation, highlighting the need for microbiota-targeted interventions	Potential for probiotics in asthma prevention and respiratory infection control
Fanos et al., 2020 [26]	Narrative Review	Lung microbiota, gut-lung axis, metabolomics, COVID-19, probiotics	Acute Respiratory Distress Syndrome (ARDS), pneumonia, viral infections, chronic lung diseases	Lung microbiota composition is crucial in acute and chronic respiratory diseases. Gut bacteria translocate to the lungs in ARDS, worsening inflammation and outcomes.	Microbiota alterations contribute to respiratory diseases; potential for microbiome-targeted therapies.	Potential for microbiome-targeted therapies in ICU settings and respiratory disease management.

				Probiotics and diet interventions may modulate gut-lung interactions.		
Dera et al., 2025 [27]	Narrative Review	Gut microbiota, maternal microbiota, immune system development, allergic diseases, asthma	Asthma, allergic rhinitis, atopic dermatitis, food allergies	Early gut microbiota plays a critical role in shaping immune responses and preventing allergic diseases. Maternal microbiota composition and interventions like probiotics may influence immune development.	Maternal and neonatal microbiota influence immune tolerance and respiratory disease risk.	Potential for microbiota-targeted interventions in pregnancy and early life to prevent allergies and asthma.
Marathe et al., 2022 [28]	Narrative Review	Airway microbiome, gut-lung axis, diet, environment, racial disparities in asthma	Asthma prevalence, severity, immune modulation, airway inflammation	Asthma is influenced by environmental, dietary, and microbial factors, particularly in high-risk populations. Targeting the microbiota through diet, probiotics,	Microbial dysbiosis affects immune balance, inflammation, and asthma severity.	Potential for microbiota-based interventions to improve asthma outcomes, especially in underserved populations.

				and therapeutics may offer new treatment strategies.		
Zhou et al., 2021 [29]	Narrative Review	Gut microbiota, lung microbiota, gut-lung axis, immune modulation, probiotics, SCFAs	Asthma, tuberculosis, lung cancer, COPD, pneumonia	The gut-lung axis plays a key role in lung health, with microbial dysbiosis influencing immune regulation and respiratory disease progression. Modulation of gut microbiota via diet, probiotics, and microbiome-targeted therapies presents a potential strategy for lung disease management.	Microbial dysbiosis alters immune homeostasis and inflammation, contributing to respiratory diseases.	Potential for microbiota-based interventions in asthma, COPD, and lung cancer management.
Rowland et al., 2024 [30]	Review & Opinion	Short-chain fatty acids (SCFAs) - Acetate, Propionate, Butyrate	Respiratory infections, allergic airway diseases, lung immune function	SCFA levels influence the gut-lung axis, with potential protective effects against infections and	SCFAs can be targeted as potential interventions to improve respiratory health, particularly in vulnerable populations (e.g., hospitalized	SCFA supplementation or dietary modulation could serve as a therapeutic approach to respiratory conditions.

				allergic airway diseases.	patients, children, athletes).	
Menegati et al., 2023 [31]	Narrative Review	Gut and lung microbiota, obesity-related dysbiosis, maternal obesity impact on microbiome	Asthma severity, obesity-related asthma phenotypes, lung function impairment	Obesity modifies immune responses in asthma, exacerbating inflammation and reducing treatment effectiveness. Gut-lung axis disturbances contribute to disease severity.	Supports the role of gut and lung microbiota in asthma pathogenesis, particularly in obesity-related cases.	Highlights microbiota-targeted interventions (e.g., SCFA supplementation, dietary modifications) as potential therapeutic strategies for asthma.
Scaldaferri et al., 2020 [32]	Narrative Review	SARS-CoV-2 impact on gut microbiota, gut-lung axis, fecal-oral transmission hypothesis	COVID-19 respiratory severity, immune response modulation, cytokine storm	The gut-lung axis may contribute to COVID-19 severity through immune dysregulation and microbiota alterations.	Microbiota-targeted interventions, such as probiotics or diet, could modulate COVID-19 outcomes and immune responses.	Suggests microbiome-based therapies as potential adjunct treatments for COVID-19 complications.
Esposito & Principi, 2017 [33]	Narrative Review	Nasopharyngeal microbiota composition, colonization resistance, microbial interactions, probiotic interventions	Respiratory tract infections, asthma, microbiota modulation	Modulating nasopharyngeal microbiota through probiotics and prebiotics could reduce respiratory disease incidence.	Supports the role of the upper respiratory microbiota in preventing respiratory diseases and its potential interaction with gut microbiota.	Suggests probiotic use as a preventive strategy for pediatric respiratory diseases.
Aslam et al., 2024 [34]	Systematic Review	Gut microbiota diversity,	Atopic wheeze, asthma	Gut microbiota compositio	Findings reinforce the role of early-	Potential for early screening of

		microbial composition (bacteria and fungi), and asthma risk	development in childhood	n in early life is linked to asthma development; interventions like probiotics and dietary modifications may reduce risk.	life microbiota in shaping immune responses and influencing asthma risk.	microbiota composition as a predictive tool for asthma risk; microbiota-targeted therapies could aid asthma prevention.
Valverde-Molina & García-Marcos, 2023 [35]	Narrative Review	Gut and airway microbiota, microbial dysbiosis, early-life colonization, bacterial metabolites, microbiome-based interventions	Asthma onset, severity, phenotypes, persistence, immune system interactions	Microbial dysbiosis in early life is strongly linked to asthma development and persistence; interventions targeting microbiota composition may influence disease outcomes.	Microbiota modulation (probiotics, diet, bacterial lysates) could serve as preventive or therapeutic approaches for asthma.	Suggests microbiome-targeted therapies (diet, probiotics, bacterial lysates) for asthma prevention and management.
Wolska et al., 2024 [36]	Narrative Review	Gut microbiota composition, immune system immaturity, microbiota-targeted interventions (probiotics, prebiotics, fecal microbiota transplantation)	Asthma, chronic obstructive pulmonary disease (COPD), bronchopulmonary dysplasia (BPD)	Gut dysbiosis in preterm infants contributes to long-term respiratory health risks, highlighting the importance of microbiota-targeted	Microbiota modulation through probiotics, prebiotics, and FMT could improve immune development and reduce respiratory disease risk in preterm infants.	Supports early-life microbiota interventions to reduce respiratory disease burden in preterm infants.

				interventions.		
Durack & Christophersen, 2020 [37]	Narrative Review	Gut and airway microbiota in pediatric respiratory health	Asthma, recurrent wheezing, acute respiratory illness (ARI)	Dysbiosis in gut and airway microbiomes linked to increased respiratory disease risk	Early-life microbiome perturbations influence immune responses and respiratory disease susceptibility	Supports microbiome-targeted interventions for respiratory disease prevention
Di Gangi et al., 2020 [38]	Mini Review	Gut Microbiota, T-Cell Response, Clostridium spp, Bacteroides spp, SCFAs, Fungal Dysbiosis	Allergic Asthma (AAS), T-Cell Polarization (Th2, Th9, Th17, Tregs), Lung Inflammation	Gut Microbiota Influences Allergic Asthma via T-Cell Modulation; SCFAs Enhance Tregs and Reduce Th2/Th9 Activity; Antibiotic-Induced Dysbiosis Exacerbates Asthma	Supports a Strong Gut-Lung Connection; SCFAs and Microbial Modulation as Potential Therapeutic Targets for Asthma Prevention	Potential for Microbiota-Targeted Therapies (Probiotics, SCFAs, Antibiotic Stewardship) in Asthma Management
Kalbermatter et al., 2021 [39]	Narrative Review	Maternal microbiota, early life colonization, breast milk	Neonatal immune development, susceptibility to respiratory diseases	Maternal microbiota, early life colonization, and breast milk intake are critical for newborn immune system development	Microbiota-driven immune priming influences lung immunity and long-term respiratory health	Insights for microbiota-targeted therapies in neonates; potential for probiotic and maternal dietary interventions
Hu et al., 2023 [40]	Narrative Review	Gut microbiota dysbiosis, lipid metabolism, chronic low-grade	Asthma severity, immune response, metabolic dysfunction	Gut microbiota acts as a bridge between obesity and asthma, influencing	Potential for microbiota-targeted therapies in managing obesity-related asthma	Supports probiotic and dietary interventions for childhood obesity and asthma

		inflammation		immune responses and metabolic pathways		
Suárez-Martínez et al., 2024 [41]	Narrative Review	Gut microbiota dysbiosis, maternal diet, delivery mode, antibiotic exposure, infant feeding	Asthma onset, immune system development, respiratory inflammation	Early gut microbiota dysbiosis can promote asthma through immune modulation, with diet and delivery mode playing critical roles	Targeting gut microbiota in early life could reduce asthma risk by enhancing immune homeostasis	Supports microbiota-targeted strategies (probiotics, dietary interventions) to prevent childhood asthma
Atto et al., 2024 [42]	Narrative Review	Respiratory microbiome, gut-lung axis, microbial interactions	Chronic suppurative lung disease (CSLD), bronchiectasis progression, inflammation	Chronic wet cough in children is strongly associated with an altered airway microbiome and persistent bacterial infections, which drive inflammation and lung damage	Dysbiosis in the respiratory and gut microbiota may modulate immune responses and disease progression in CSLD	Highlights potential for microbiota-targeted therapies, probiotics, and novel diagnostics in pediatric chronic lung diseases
Valentino et al., 2022 [43]	Narrative Review	Gut microbiota dysbiosis, SARS-CoV-2 interaction, immune modulation	COVID-19 severity, immune response, gut-lung axis dysfunction	Gut microbiota dysbiosis in children with COVID-19 may impact immune responses and disease	Microbiota-based interventions may modulate COVID-19 severity and immune recovery in children	Potential for probiotics and microbiota-targeted therapies to mitigate COVID-19 effects in children

				progression		
Chung et al., 2017a [44]	Review (JACI)	Gut and lung microbiota dysbiosis, early-life microbial exposure, antibiotic effects	Asthma onset, severity, immune dysregulation	Asthma is linked to airway microbial imbalances; gut microbiota may shape lung immune responses via the gut-lung axis	Targeting gut and airway microbiota could help modulate asthma severity and progression	Supports microbiota-targeted therapies, probiotics, and dietary interventions for asthma prevention
Azad & Kozyrskyj, 2012 [45]	Narrative Review	Perinatal gut microbiota programming, birth mode, breastfeeding, antibiotics, probiotics, stress	Asthma onset, immune system modulation, inflammation	Early-life gut microbiota disruptions can contribute to asthma risk through immune dysregulation	Microbiota-targeted strategies (probiotics, dietary interventions) could mitigate asthma risk	Supports microbiota-based interventions for asthma prevention
Kanj & Skalski, 2024 [46]	Narrative Review	Gut Mycobiome, Fungal Dysbiosis, Candida Overgrowth	Asthma Severity, Allergic Airway Inflammation, Immune Modulation	Gut fungal dysbiosis contributes to asthma severity through immune system modulation.	Targeting gut mycobiome could offer therapeutic approaches for asthma.	Potential for antifungal and probiotic therapies targeting gut mycobiome.
Meirlaen et al., 2022 [47]	Narrative Review	Probiotics, Gut Microbiota, Gut-Lung Axis	Asthma Prevention, Allergic Rhinitis, Immune Modulation	Probiotic supplementation may benefit allergic diseases but lacks strong evidence for asthma prevention or treatment.	Probiotic interventions could help regulate immune responses and reduce allergic inflammation.	Potential adjunct therapy for allergic diseases; strain-specific effects require further research.

Toldi et al., 2021 [48]	Narrative Review	T lymphocytes, microbiome, metabolome, hyperoxia, corticosteroids, gut-lung axis	Bronchopulmonary dysplasia (BPD), lung inflammation, immune responses	BPD is associated with immune dysregulation, microbiome changes, and inflammation influenced by multiple factors	Gut microbiome influences lung immunity and BPD development; perinatal antibiotic exposure impacts disease risk	Insights into immune mechanisms and microbiome interactions can guide interventions in BPD
Saeed et al., 2022 [49]	Narrative Review	Gut microbiota alterations in childhood diseases, dysbiosis	Asthma, allergic rhinitis, cystic fibrosis progression	Gut microbiota alterations contribute to immune disorders, allergic diseases, and lung conditions	Supports the gut-lung connection in inflammatory and allergic disorders	Microbiota-targeted interventions may help prevent childhood diseases
Wang et al., 2023 [50]	Narrative Review	Probiotic interventions targeting gut-lung axis	Influenza severity, upper respiratory tract infections (URTIs)	Probiotics show potential in reducing influenza risk and severity through immune regulation	Supports gut microbiota role in pulmonary immune responses and infection resistance	Probiotic-based interventions could be an adjunct strategy for respiratory infections
Manos, 2022 [51]	Narrative Review / Foundational	Gut, lung, skin, oral, brain microbiomes; axes of communication (gut-lung, gut-brain, gut-skin)	Discusses asthma, CF, COPD, pneumonia, tuberculosis in microbiota context	Dysbiosis in gut can influence distal organs via gut-lung, gut-brain axes; microbial communication key to disease development	Highlights systemic impact of microbiota; gut dysbiosis associated with lung pathology in asthma, CF, TB	Supports holistic microbiome understanding; indirect pediatric relevance; foundational backgro
Zhang et al., 2023 [52]	Perspective Review	Gut microbiota dysbiosis,	Respiratory symptoms, cytokine	Gut microbiota influences	Strong gut-lung axis component:	High; supports use of probiotics,

		SCFA metabolism, tryptophan metabolism, ACE2 modulation	storm, DLCO, long COVID with respiratory dysfunction	COVID-19 severity, recovery, and vaccine response; dysbiosis linked to long COVID symptoms	dysbiosis impacts immune lung function, inflammation, and respiratory outcomes	prebiotics, FMT; links to long COVID and pediatric impact
Verstegen et al., 2021 [53]	Narrative Review	Dietary fibers (soluble/insoluble); fermentation; SCFA production; gut microbiota modulation	Allergic asthma, airway hyperresponsiveness (AHR), lung inflammation, cytokines (IL-4, IL-5, IL-13)	Dietary fibers impact gut microbiota, increase SCFA, reduce allergic lung inflammation via immune modulation	Strong theoretical and mechanistic support for gut-lung axis involvement in asthma prevention/management	High; supports dietary modulation of microbiota for early prevention of allergic diseases in children
Chung, 2017 b [54]	Narrative Review	Lung microbiota, gut microbiota, gut-lung axis, dysbiosis	Asthma phenotypes, severity, corticosteroid resistance, exacerbations	Lung and gut dysbiosis are associated with asthma onset, severity, and phenotypes; mechanistic links proposed	Strong; reviews evidence for gut-lung crosstalk affecting asthma via immune pathways	High; informs clinical phenotyping and microbiota-based interventions

1. Hufnagl K., Pali-Schöll I., Roth-Walter F, Jensen-Jarolim E. Dysbiosis of the gut and lung microbiome has a role in asthma. Semin Immunopathol. febbraio 2020;42(1):75–93.

2. Kloepfer K.M., Kennedy J.L. Childhood respiratory viral infections and the microbiome. J Allergy Clin Immunol. ottobre 2023;152(4):827–34.

3. Yuksel N., Gelmez B, Yildiz-Pekoz A. Lung Microbiota: Its Relationship to Respiratory System Diseases and Approaches for Lung-Targeted Probiotic Bacteria Delivery. Mol Pharm. 3 luglio 2023;20(7):3320–37.

4. Perdijk O, Azzoni R, Marsland BJ. The microbiome: an integral player in immune homeostasis and inflammation in the respiratory tract. Physiol Rev. 1 aprile 2024;104(2):835–79.

5. Willis KA, Siefker DT, Aziz MM, White CT, Mussarat N, Gomes CK, et al. Perinatal maternal antibiotic exposure augments lung injury in offspring in experimental bronchopulmonary dysplasia. Am J Physiol Lung Cell Mol Physiol. 1 febbraio 2020;318(2):L407–18.

6. Yang S, Qiao L, Shi J, Xie L, Liu Y, Xiong Y, et al. Clinical Study of Correlation for the Intestinal and Pharyngeal Microbiota in the Premature Neonates. Front Pediatr [Internet]. 16 febbraio 2021 [citato 21 maggio 2025];9. Disponibile su: https://www.frontiersin.org/journals/pediatrics/articles/10.3389/fped.2021.632573/full

7. Ranucci G, Buccigrossi V, de Freitas MB, Guarino A, Giannattasio A. Early-Life Intestine Microbiota and Lung Health in Children. J Immunol Res. 2017;2017:8450496.

8. Kahhaleh FG, Barrientos G, Conrad ML. The gut-lung axis and asthma susceptibility in early life. Acta Physiol Oxf Engl. marzo 2024;240(3):e14092.

9. Xue S, Abdullahi R, Wu N, Zheng J, Su M, Xu M. Gut microecological regulation on bronchiolitis and asthma in children: A review. Clin Respir J. ottobre 2023;17(10):975–85.

10. Zhu W, Wu Y, Liu H, Jiang C, Huo L. Gut–Lung Axis: Microbial Crosstalk in Pediatric Respiratory Tract Infections. Front Immunol [Internet]. 18 novembre 2021 [citato 22 maggio 2025];12:741233. Disponibile su: https://www.ncbi.nlm.nih.gov/pmc/articles/PMC8637285/

11. Piersigilli F, Van Grambezen B, Hocq C, Danhaive O. Nutrients and Microbiota in Lung Diseases of Prematurity: The Placenta-Gut-Lung Triangle. Nutrients. 13 febbraio 2020;12(2):469.

12. Zama D, Totaro C, Biscardi L, Rocca A, Turroni S, Brigidi P, et al. The Relationship between Gut Microbiota and Respiratory Tract Infections in Childhood: A Narrative Review. Nutrients. 21 luglio 2022;14(14):2992.

13. Pammi M, Lal CV, Wagner BD, Mourani PM, Lohmann P, Luna RA, et al. Airway Microbiome and Development of Bronchopulmonary Dysplasia in Preterm Infants: A Systematic Review. J Pediatr. gennaio 2019;204:126-133.e2.

14. Stricker S, Hain T, Chao CM, Rudloff S. Respiratory and Intestinal Microbiota in Pediatric Lung Diseases-Current Evidence of the Gut-Lung Axis. Int J Mol Sci. 18 giugno 2022;23(12):6791.

15. Tirone C, Pezza L, Paladini A, Tana M, Aurilia C, Lio A, et al. Gut and Lung Microbiota in Preterm Infants: Immunological Modulation and Implication in Neonatal Outcomes. Front Immunol. 2019;10:2910.

16. Gao Y, Wang K, Lin Z, Cai S, Peng A, He L, et al. The emerging roles of microbiome and short-chain fatty acids in the pathogenesis of bronchopulmonary dysplasia. Front Cell Infect Microbiol [Internet]. 20 settembre 2024 [citato 22 maggio 2025];14. Disponibile su:

https://www.frontiersin.org/journals/cellular-and-infection-microbiology/articles/10.3389/fcimb.2024.1434687/full

17. Colombo SFG, Nava C, Castoldi F, Fabiano V, Meneghin F, Lista G, et al. Preterm Infants' Airway Microbiome: A Scoping Review of the Current Evidence. Nutrients. 6 febbraio 2024;16(4):465.

18. Campbell S, Gerasimidis K, Milling S, Dicker AJ, Hansen R, Langley RJ. The lower airway microbiome in paediatric health and chronic disease. Paediatr Respir Rev. dicembre 2024;52:31–43.

19. Chan CKY, Tao J, Chan OS, Li HB, Pang H. Preventing Respiratory Tract Infections by Synbiotic Interventions: A Systematic Review and Meta-Analysis of Randomized Controlled Trials. Adv Nutr Bethesda Md. 1 luglio 2020;11(4):979–88.

20. Meirlaen L, Levy EI, Vandenplas Y. Prevention and Management with Pro-, Pre and Synbiotics in Children with Asthma and Allergic Rhinitis: A Narrative Review. Nutrients. 14 marzo 2021;13(3):934.

21. Pirr S, Willers M, Viemann D. The neonate respiratory microbiome. Acta Physiol [Internet]. 2025 [citato 22 maggio 2025];241(2):e14266. Disponibile su: https://onlinelibrary.wiley.com/doi/abs/10.1111/apha.14266

22. Musso P, Chiappini E, Bernardini R. Human Microbiome and Allergic Diseases in Children: Pathogenetic Role and Therapeutic Options. Curr Pediatr Rev. 2020;16(2):89–94.

23. Yang K, Dong W. Perspectives on Probiotics and Bronchopulmonary Dysplasia. Front Pediatr. 2020;8:570247.

24. Sun T, Yu H, Fu J. Respiratory Tract Microecology and Bronchopulmonary Dysplasia in Preterm Infants. Front Pediatr. 2021;9:762545.

25. Pulvirenti G, Parisi GF, Giallongo A, Papale M, Manti S, Savasta S, et al. Lower Airway Microbiota. Front Pediatr [Internet]. 27 settembre 2019 [citato 22 maggio 2025];7:393. Disponibile su: https://www.ncbi.nlm.nih.gov/pmc/articles/PMC6776601/

26. Fanos V, Pintus MC, Pintus R, Marcialis MA. Lung microbiota in the acute respiratory disease: from coronavirus to metabolomics. J Pediatr Neonatal Individ Med JPNIM [Internet]. 13 aprile 2020 [citato 22 maggio 2025];9(1):e090139–e090139. Disponibile su: https://jpnim.com/index.php/jpnim/article/view/090139

27. Dera N, Kosińska-Kaczyńska K, Żeber-Lubecka N, Brawura-Biskupski-Samaha R, Massalska D, Szymusik I, et al. Impact of Early-Life Microbiota on Immune System Development and Allergic Disorders. Biomedicines [Internet]. 7 gennaio 2025 [citato 22 maggio 2025];13(1):121. Disponibile su: https://www.ncbi.nlm.nih.gov/pmc/articles/PMC11762082/

28. Marathe SJ, Snider MA, Flores-Torres AS, Dubin PJ, Samarasinghe AE. Human matters in asthma: Considering the microbiome in pulmonary health. Front Pharmacol [Internet]. 2 dicembre 2022 [citato 22 maggio 2025];13:1020133. Disponibile su: https://www.ncbi.nlm.nih.gov/pmc/articles/PMC9755222/

29. Zhou A, Lei Y, Tang L, Hu S, Yang M, Wu L, et al. Gut Microbiota: the Emerging Link to Lung Homeostasis and Disease. J Bacteriol. 25 gennaio 2021;203(4):e00454-20.

30. Rowland SN, Green CG, Halliwill JR, Singanayagam A, Heaney LM. Gut feelings on short-chain fatty acids to regulate respiratory health. Trends Endocrinol Metab [Internet]. 4 gennaio 2025 [citato 22 maggio 2025]; Disponibile su: https://www.sciencedirect.com/science/article/pii/S1043276024003291

31. Menegati LM, de Oliveira EE, Oliveira B de C, Macedo GC, de Castro E Silva FM. Asthma, obesity, and microbiota: A complex immunological interaction. Immunol Lett. marzo 2023;255:10–20.

32. Scaldaferri F, Ianiro G, Privitera G, Lopetuso LR, Vetrone LM, Petito V, et al. The Thrilling Journey of SARS-CoV-2 into the Intestine: From Pathogenesis to Future Clinical Implications. Inflamm Bowel Dis. 20 agosto 2020;26(9):1306–14.

33. Esposito S, Principi N. Impact of nasopharyngeal microbiota on the development of respiratory tract diseases. Eur J Clin Microbiol Infect Dis Off Publ Eur Soc Clin Microbiol. gennaio 2018;37(1):1–7.

34. Aslam R, Herrles L, Aoun R, Pioskowik A, Pietrzyk A. Link between gut microbiota dysbiosis and childhood asthma: Insights from a systematic review. J Allergy Clin Immunol Glob. agosto 2024;3(3):100289.

35. Valverde-Molina J, García-Marcos L. Microbiome and Asthma: Microbial Dysbiosis and the Origins, Phenotypes, Persistence, and Severity of Asthma. Nutrients. 17 gennaio 2023;15(3):486.

36. Wolska M, Wypych TP, Rodríguez-Viso P. The Influence of Premature Birth on the Development of Pulmonary Diseases: Focus on the Microbiome. Metabolites [Internet]. luglio 2024 [citato 22 maggio 2025];14(7):382. Disponibile su: https://www.mdpi.com/2218-1989/14/7/382

37. Durack J, Christophersen CT. Human Respiratory and Gut Microbiomes—Do They Really Contribute to Respiratory Health? Front Pediatr [Internet]. 3 settembre 2020 [citato 22 maggio 2025];8. Disponibile su: https://www.frontiersin.org/journals/pediatrics/articles/10.3389/fped.2020.00528/full

38. Di Gangi A, Di Cicco ME, Comberiati P, Peroni DG. Go With Your Gut: The Shaping of T-Cell Response by Gut Microbiota in Allergic Asthma. Front Immunol. 2020;11:1485.

39. Kalbermatter C, Fernandez Trigo N, Christensen S, Ganal-Vonarburg SC. Maternal Microbiota, Early Life Colonization and Breast Milk Drive Immune Development in the Newborn. Front Immunol [Internet]. 13 maggio 2021 [citato 22 maggio 2025];12. Disponibile su: https://www.frontiersin.org/journals/immunology/articles/10.3389/fimmu.2021.683022/full

40. Hu M, Zhao X, Liu Y, Zhou H, You Y, Xue Z. Complex interplay of gut microbiota between obesity and asthma in children. Front Microbiol [Internet]. 3 novembre 2023 [citato 22 maggio 2025];14:1264356. Disponibile su: https://www.ncbi.nlm.nih.gov/pmc/articles/PMC10655108/

41. Suárez-Martínez C, Santaella-Pascual M, Yagüe-Guirao G, García-Marcos L, Ros G, Martínez-Graciá C. The Early Appearance of Asthma and Its Relationship with Gut Microbiota: A Narrative Review. Microorganisms [Internet]. luglio 2024 [citato 22 maggio 2025];12(7):1471. Disponibile su: https://www.mdpi.com/2076-2607/12/7/1471

42. Atto B, Anteneh Y, Bialasiewicz S, Binks MJ, Hashemi M, Hill J, et al. The Respiratory Microbiome in Paediatric Chronic Wet Cough: What Is Known and Future Directions. J Clin Med. 28 dicembre 2023;13(1):171.

43. Valentino MS, Esposito C, Colosimo S, Caprio AM, Puzone S, Guarino S, et al. Gut microbiota and COVID-19: An intriguing pediatric perspective. World J Clin Cases. 16 agosto 2022;10(23):8076–87.

44. Chung KF. Airway microbial dysbiosis in asthmatic patients: A target for prevention and treatment? J Allergy Clin Immunol. aprile 2017;139(4):1071–81.

45. Azad MB, Kozyrskyj AL. Perinatal programming of asthma: the role of gut microbiota. Clin Dev Immunol. 2012;2012:932072.

46. Kanj AN, Skalski JH. Gut Mycobiome and Asthma. J Fungi [Internet]. marzo 2024 [citato 22 maggio 2025];10(3):192. Disponibile su: https://www.mdpi.com/2309-608X/10/3/192

47. Meirlaen L, Levy EI, Vandenplas Y. Chapter 10 - Probiotics in the prevention and management of allergic diseases (asthma and allergic rhinitis). In: Dwivedi MK, Amaresan N, Sankaranarayanan A, Kemp EH, curatori. Probiotics in the Prevention and Management of Human Diseases [Internet]. Academic Press; 2022 [citato 22 maggio 2025]. p. 139–46. Disponibile su: https://www.sciencedirect.com/science/article/pii/B9780128237335000271

48. Toldi G, Hummler H, Pillay T. T Lymphocytes, Multi-Omic Interactions and Bronchopulmonary Dysplasia. Front Pediatr. 2021;9:694034.

49. Saeed NK, Al-Beltagi M, Bediwy AS, El-Sawaf Y, Toema O. Gut microbiota in various childhood disorders: Implication and indications. World J Gastroenterol. 14 maggio 2022;28(18):1875–901.

50. Wang YH, Limaye A, Liu JR, Wu TN. Potential probiotics for regulation of the gut-lung axis to prevent or alleviate influenza in vulnerable populations. J Tradit Complement Med [Internet]. 1 marzo 2023 [citato 22 maggio 2025];13(2):161–9. Disponibile su: https://www.sciencedirect.com/science/article/pii/S2225411022000608

51. Manos J. The human microbiome in disease and pathology. APMIS Acta Pathol Microbiol Immunol Scand. dicembre 2022;130(12):690–705.

52. Zhang F, Lau RI, Liu Q, Su Q, Chan FKL, Ng SC. Gut microbiota in COVID-19: key microbial changes, potential mechanisms and clinical applications. Nat Rev Gastroenterol Hepatol. maggio 2023;20(5):323–37.

53. Verstegen REM, Kostadinova AI, Merenciana Z, Garssen J, Folkerts G, Hendriks RW, et al. Dietary Fibers: Effects, Underlying Mechanisms and Possible Role in Allergic Asthma Management. Nutrients. 19 novembre 2021;13(11):4153.

54. Chung KF. Potential Role of the Lung Microbiome in Shaping Asthma Phenotypes. Ann Am Thorac Soc. novembre 2017;14(Supplement_5):S326–31.
